# Toxoplasmosis – A Global Threat. Correlation of Latent Toxoplasmosis with Specific Disease Burden in a Set of 88 Countries

**DOI:** 10.1371/journal.pone.0090203

**Published:** 2014-03-24

**Authors:** Jaroslav Flegr, Joseph Prandota, Michaela Sovičková, Zafar H. Israili

**Affiliations:** 1 Department of Biology, Faculty of Science, Charles University in Prague, Prague, Czech Republic; 2 Department of Social Pediatrics, Faculty of Health Sciences, Wroclaw Medical University, Wroclaw, Poland; 3 Department of Medicine, Emory University School of Medicine, Atlanta, Georgia, United States of America; National Institute of Medical Research, United Kingdom

## Abstract

**Background:**

Toxoplasmosis is becoming a global health hazard as it infects 30–50% of the world human population. Clinically, the life-long presence of the parasite in tissues of a majority of infected individuals is usually considered asymptomatic. However, a number of studies show that this ‘asymptomatic infection’ may also lead to development of other human pathologies.

**Aims of the Study:**

The purpose of the study was to collect available geoepidemiological data on seroprevalence of toxoplasmosis and search for its relationship with mortality and disability rates in different countries.

**Methods and Findings:**

Prevalence data published between 1995–2008 for women in child-bearing age were collected for 88 countries (29 European). The association between prevalence of toxoplasmosis and specific disease burden estimated with age-standardized Disability Adjusted Life Year (DALY) or with mortality, was calculated using General Linear Method with Gross Domestic Product per capita (GDP), geolatitude and humidity as covariates, and also using nonparametric partial Kendall correlation test with GDP as a covariate. The prevalence of toxoplasmosis correlated with specific disease burden in particular countries explaining 23% of variability in disease burden in Europe. The analyses revealed that for example, DALY of 23 of 128 analyzed diseases and disease categories on the WHO list showed correlations (18 positive, 5 negative) with prevalence of toxoplasmosis and another 12 diseases showed positive trends (p<0.1). For several obtained significant correlations between the seroprevalence of toxoplasmosis and specific diseases/clinical entities, possible pathophysiological, biochemical and molecular explanations are presented.

**Conclusions:**

The seroprevalence of toxoplasmosis correlated with various disease burden. Statistical associations does not necessarily mean causality. The precautionary principle suggests however that possible role of toxoplasmosis as a triggering factor responsible for development of several clinical entities deserves much more attention and financial support both in everyday medical practice and future clinical research.

## Introduction

Toxoplasmosis, a disease caused by the obligate apicomplexan intracellular protozoan *Toxoplasma gondii*, is one of the world's most common parasites infecting most genera of warm-blooded animals (more than 30 species of birds and 300 species of mammals). It is the most prevalent infection in humans (estimated to be 30–50% of the world population), more than latent tuberculosis which infects about one-third of the human population (WHO, www.who.int/entity/tb/publications/2009/tbfactsheet_2009update_one_page.pdf, accessed July 2013). The definitive hosts are representatives of the felid (cat) family. Nicolle and Manceaux (1908) first observed the parasites in the blood and tissues of a North African rodent, *Ctenodactylus gondii*, and named it *Toxoplasma* (arclike form) *gondii* (after the rodent host) [1]. There are three infective stages of *T. gondii*: a) a rapidly dividing invasive tachyzoite; b) a slowly dividing bradyzoite in tissue cysts, which can persist inside human cells for protracted periods; and c) an environmental stage, the sporozoite, protected inside an oocyst. The oocysts, remarkably stable environmentally, are transmitted to other hosts through inadvertent ingestion.

### Seroprevalence of toxoplasmosis

Seroprevalence is a measure of the accumulated exposure during a person's lifetime in a particular social setting. Most of the more than one third of the world's human population who are infected with *T. gondii* remain asymptomatic because the immune system usually keeps the parasite from causing illness. Chronic, usually lifelong, infection with *Toxoplasma* that is not accompanied with overt clinical symptoms of toxoplasmosis disease is termed latent toxoplasmosis while chronic infection associated with continuous or recurrent clinical symptoms is termed chronic toxoplasmosis (this form of disease is relatively rare in Europe and Northern America). Worldwide seroprevalence of the parasite measured by specific anti-*Toxoplasma* IgG antibodies varies between 1% and 100% depending on the environmental and socioeconomic conditions, including eating habits and health-related practices [2]–[5], general level of hygiene, host susceptibility, geographical location (geolatitude) and humidity of the soil. The incidence of infection is higher in warmer and humid climates and increases with age [5]. The lowest seroprevalence (∼1%) was found in some countries in the Far East and the highest (>90%) in some parts of European and South American countries. In the United States, the Centers for Disease Control and Prevention (CDC) reported an overall seroprevalence of 11% [National Health and Nutritional Examination Survey between 1999 and 2004]; another survey reported a higher number (22.5%) [6]. Nevertheless, toxoplasmosis is one of the leading causes of death attributed to foodborne illness [7]. In European countries, the prevalence ranges between 10% to 60%, and in some regions as high as 90% [8]. In one study, 84% of pregnant women had serum antibodies against the parasite [3]. Data from 88 countries are presented in Table 1; most of the published data on seroprevalence are in women of childbearing age and/or those who are pregnant.

**Table 1:** Prevalence of latent toxoplasmosis in women of childbearing age in various countries.

Country	Prevalence (%)	Adj. Prevalence (%)	Reference	Period	No.
Albania	49	42	[10]	2004–2005	496
Argentina	60	53	[11]	2001	1007
Australia	23	16	[12]	2001	308
Austria	42	36	[13]	1997	4601
Bahrain	22	16	[14]	2005	3499
Bangladesh	38	38	[15]	1995–1996	286
Belgium	49	42	[16]	2004	16541
Benin	54	47	[17]	1993	211
Brazil	50	50	[18]	2012	2136
Burkina Faso	25	25	[19]	2006	336
Cameroon	77	70	[20]	1992	1014
Canada	20	17	[21]	2006	NA
Colombia	54	54	[22]	2006	630
Costa Rica	76	76	[23]	1996	1234
Croatia	29	24	[24]	2000	1109
Cuba	55	55	[25]	2004	526
Czech Republic	20	16	[26]	2007	1053
Congo	60	60	[27]	1990	2897
Denmark	28	20	[28]	1999	89873
Egypt	42	36	[29]	1995	62
Estonia	68.6	45	[30]	1999–2000	1277
Ethiopia	74	66	[31]	2012	1016
Finland	20	17	[32]	1989	16733
France	54	47	[33]	1995	13459
Gabon	71	71	[34]	1997	767
Germany	63	50	[35]	1999	4854
Greece	25	21	[36]	2004	5532
Grenada	57	50	[37]	2006	534
Hungary	45	39	[38]	2000	31759
Chile	39	33	[39]	1996	7536
China	11	11	[40]	2006	235
Iceland	13	8	[30]	1998	440
India	35	35	[41]	2003	180
Indonesia	53	46	[42]	2006	17735
Iran	39	33	[43]	2007	576
Iraq	49	42	[44]	2002	254
Ireland	34	25	[45]	2008	20252
Israel	21	17	[46]	1989	213
Italy	23	16	[47]	2004	3426
Jamaica	57	57	[48]	1986	1604
Japan	10	8	[49]	2011	4466
Jordan	47	40	[50]	2005	280
Kuwait	46	53	[51]	2002–2005	225
Lebanon	62	62	[52]	2010	232
Libya	45	34	[53]	2007	143
Lithuania	40	34	[54]	1991	NA
Macedonia	22	18	[55]	2005	NA
Madagascar	84	84	[56]	1992	599
Malaysia	49	42	[57]	2003	200
Mexico	49	49	[58]	2006	NA
Montenegro	27	23	[55]	NA	NA
Morocco	51	44	[59]	2007	2456
Mozambique	19	13	[60]	2008	150
Nepal	55	55	[61]	1998	345
Netherlands	35	26	[62]	2004	7521
New Zealand	35	26	[63]	2004	500
Nigeria	78	71	[64]	1992	352
Norway	11	9	[65]	1993	35940
Pakistan	33	28	[66]	1997	105
Papua New Guinea	18	15	[67]	1990	197
Peru	39	33	[68]	NA	NA
Poland	40	34	[69]	2003	4916
Portugal	24	17	[70]	2011	401
Qatar	35	30	[71]	2005–2008	1857
South Korea	4	3	[72]	2000	NA
Romania	44	38	[73]	2008	184
Sao Tome and Principe	75	68	[74]	2007	499
Saudi Arabia	32	27	[75]	1991	921
Senegal	40	34	[76]	1993	353
Serbia	31	26	[55]	2007	765
Singapore	17	14	[77]	NA	120
Slovakia	22	18	[78]	2008	656
Slovenia	25	21	[79]	2002	21270
Spain	32	23	[80]	2004	16362
Sudan	42	36	[81]	2003	487
Sweden	18	13	[82]	2001	40978
Switzerland	35	26	[83]	2006	NA
Tanzania	35	35	[84]	1991	549
Thailand	13	11	[83]	2001	1200
Togo	75	68	[85]	1991	620
Trinidad and Tobago	43	43	[86]	2008	450
Tunisia	43	37	[87]	1996	2231
Turkey	54	47	[88]	2005	1149
United Arab Emirates	23	19	[89]	1997	1503
UK	9	6	[90]	2005	1897
USA	11	9	[91]	2007	NA
Venezuela	38	38	[92]	2006	446
Vietnam	11	9	[93]	2003	300

The second and third column show prevalence of toxoplasmosis and prevalence adjusted to a standard age of 22 years to account for variation in childbearing age in across countries (column 1) using the formula Prevalence_adj_ = 1−(1−Prevalence)∧(22/childbearing age) [9]. Column 5 shows year(s) when the study was performed and column 6 shows number of women in the sample. For Macedonia, the 2004 WHO data were not available therefore this 30^th^ European country was not included in our data set.

In the majority of the human populations, the parasite seroprevalence increases with age, and may vary by gender [6], [94]. Latitudinal variability in the geoseroprevalence of the parasite may be due to local rainy conditions (because oocysts live longer in humid conditions), and low altitude regions (especially at mid-latitudes); a north-south seroprevalence gradient has also been reported in animals [9], [95], [96].

The seroprevalence of toxoplasmosis is high in immunocompromised patients, such as those infected with human immunodeficiency virus (HIV), and transplant or cancer patients treated with immunosuppressive agents [5], [97], [98].

It may be pointed out that the different serological methods used to obtain prevalence data are not standardized, and vary in sensitivity, specificity, and predictive values. As a consequence, no two tests produce the same results in all cases, even when carried out in the same laboratory [5].

### Genotypes and virulence of *T. gondii*

*T. gondii* strains are highly diverse but only a few lineages are widely spread. Different genotypes of the parasite show great diversity in pathogenicity and drug sensitivity. Some atypical strains have also been detected. In Europe, North America, and Africa, there are three dominant clonal lineages of *T. gondii* called type I (RH, GT1,CAST), type II (ME49, WIL, HART), and type III (VEG, MOO, SOU), as well as many atypical genotypes which differ in prevalence, virulence, migratory capacity within the host, and ability to convert to the bradyzoite cyst phase [99]–[101]. Different strains of the parasite induce different cytokine responses [102], thus triggering development of various clinical and biochemical disturbances in the host, including modulation of the host cell proteome [103], [104]. Mice fed as few as 1 oocyst of *T. gondii* serotype I and several atypical strains died of acute toxoplasmosis within 21 days post inoculation, while some *T. gondii* type II, and III strains were less virulent [105]. In North America, the parasite serotype II and NE-II causes congenital toxoplasmosis, while prematurity and severity of disease at birth was associated with the coccidian NE-II serotype [106]. This serotype was also associated with rural residence, lower socioeconomic status and Hispanic ethnicity (P<0.01–0.001) [106]. A greater variety of genotypes are found in South America and Africa than in North America and Europe [107], [108], suggesting that in these continents sexual replication of the parasite occurs more frequently than in any other part of the world [109]. This genetic divergence may contribute to the higher prevalence of seropositivity and ocular disease due to *T. gondii*, as exemplified by the higher prevalence of toxoplasmosis and *Toxoplasma*-induced eye disease in southern Brazil than in any other part of the world [110].

### Transmission of *T. gondii*

Animals are infected by eating infected animals, by ingestion of or coming in contact with feces of an infected cat, or by transmission from mother to fetus. In humans, cats are the primary source of infection (contact with fecal material), but other pets may also be the secondary source of infection [3], [111], [112]. The seroprevalence of toxoplasmosis in the Arctic region proves that *T. gondii* can thrive in the absence of cats [113].

Contact with raw meat of infected animals, especially pork, is a more significant source of human infections in some countries, such as in Poland, where the majority of pigs, cattle and sheep (approximately 80%) test positive for *T. gondii* [8], [114]. Transmission of the parasite can also occur by drinking municipal/well unboiled and unbottled water containing oocysts, exposure to contaminated soil, contaminated milk, exposure of children playing in sandpits, geophagia [115], [116], eating raw or undercooked meat, especially venison [117] or rabbits [118], raw oysters, clams, or mussels [119], consumption of unwashed raw fruits and vegetables contaminated with the oocytes [117], blood transfusion [120]–[122], maternal-fetal passage of blood cells (including placental trophoblasts) [123], [124], solid organ allografts [125], [126], bone marrow transplantation [127], allogeneic stem cell transplantation [128], sputum [129], breast milk [130], [131], and semen [132] (thus, probably the infection may be transmitted via both vaginal and oral sex, significantly more frequently from seropositive to passive sex partner than vice-versa (P<0.001) [133]). Poor hygiene, lower socioeconomic status and less education, as well as exposure to certain strains of *T. gondii* may also contribute to a higher rate of infection [134].

### Cellular mechanism(s) of infection

*T. gondii* is remarkable in its ability to invade a wide variety of host cells. Invasion is an active process relying on parasite motility and the sequential secretion of proteins from secretory organelles, the micronemes, rhoptries, and the dense granules. *T. gondii* can invade and multiply inside any nucleated cell type including epithelial cells and blood leukocytes [135]. A preference to infect and multiply inside myeloid cells *in vitro* has also been reported [136], and several studies in mice indicate that the dendritic cells as well as monocytes/macrophages function as systemic parasite transporters (“Trojan horses”) during infection [137]–[142]. The parasite can be transmitted from infected dendritic cells to NK cells [143], and thus, low levels of NK cells found in pregnant women may suggest transmission of the parasite [144]. Differential infectivity and division rate of intracellular tachyzoites in human peripheral blood leukocytes and other primary human cells *in vitro* has been demonstrated depending on the cell characteristics [136].

### Clinical manifestations of toxoplasmosis

It is believed that the majority of immunocompetent individuals infected with *T*. *gondii* remain asymptomatic or have a subclinical course with minor symptoms [145]. It is nevertheless the most common food-borne parasitic infection requiring hospital treatment [146], and the third most common cause of hospitalization due to food-borne infection [147]. Both competent and immunocompromised persons can develop the disease, especially retinochoroiditis (ocular toxoplasmosis) [2], [145], [148]. In non-pregnant immunocompetent adults, acute disease may also lead to impaired eye sight [149], [150]. For example, in the United States, one million new infections occur each year, which result in approximately 20 000 cases of retinal pathology [151]. Primary infection in pregnant women is a matter of great concern, since it can be transmitted to the fetus leading to spontaneous abortion or stillbirth. A newborn exposed to *T. gondii* in utero may develop congenital toxoplasmosis with major ocular and neurological consequences. In immunosuppressed (HIV, organ transplant or cancer) patients, the infection can lead to life-threatening cerebral toxoplasmosis [97], [98], [152].

Symptomatic infection with the parasite can be categorized into four groups: 1) cervical lymphadenopathy, headache, fever, sore throat, and myalgia, with possibility of splenomegaly and brief erythematous (maculopapular) rash; 2) typhus-like exanthematous form with myocarditis, meningoencephalitis, atypical pneumonia and possibly death; 3) retinochoroiditis, which may be severe, requiring enucleation; and 4) central nervous system involvement [153]. In addition, several reports suggest that *T. gondii* infection may be responsible for additional wide range of symptoms, and development of several clinical entities (summarized in Table 2).

**Table 2:** Diseases and clinical entities associated with *T. gondii* infection.

Disease/Clinical entity	References
Congenital toxoplasmosis (encephalitis; chorioretinitis; neonatal mortality)	[100], [149], [155]–[158]
Psychosis; schizophrenia; bipolar disorder	[159]–[166]
Mood disorders; suicide; depression (?)	[167]–[174]
Obsessive-compulsive disorder	[175], [176]
Attention/concentration deficit hyperactivity disorder	[175], [177]
Anorexia	[178]–[181]
Autism spectrum disorders	[164], [177], [182]–[185]
Down's syndrome	[182], [186]–[188]
Alzheimer's disease	[182], [189]–[191]
Parkinson's disease	[192], [193]
Migraine; other headaches	[194]–[197]
Idiopathic intracranial hypertension	[177], [180], [198]
Pseudotumor cerebri	[180], [198]
Aseptic meningitis	[180], [198]
Mollaret meningitis	[199]
Epilepsy	[200], [201]
Aphasia and epilepsy (Landau-Kleffner syndrome)	[202]
Facial nerve palsy (Bell's palsy)	[203]
Hearing loss	[204], [205]
Central diabetes insipidus; syndrome of inappropriate antidiuretic hormone secretion	[156], [206]–[209]
Hypothalamo-pituitary dysfunction; panhypopituitarism	[209]–[211]
Brain tumors (meningioma; ependymoma; glioma)	[196], [212]–[216]
Non-Hodgkin's lymphoma	[217], [218]
Neoplasia	[216], [219]–[221]
Melanoma	[216], [222]–[226]
Breast cancer	[227]
Carcinoma of female genitalia, including cervical tissue	[228]
Chronic heart failure; myocarditis; arrhythmia	[229]–[231]
Inflammatory bowel disease	[232]–[234]
Ulcerative colitis	[232]
Crohn's disease	[232]
Celiac disease	[232], [235]
Abdominal hernia	[233], [236]
Hepatitis, including HCV infection	[237]–[246]
Granulomatous liver disease	[247], [248]
Liver cirrhosis; granulomatous liver disease; impaired liver function	[242]–[244], [249]–[253]
Primary biliary cirrhosis; biliary atresia; cholestatic disorders	[254]–[261]
Diabetes mellitus type 1 and 2	[189], [262]–[264]
Goitre; iodine deficiency	[265]–[268]
Hashimoto's thyroiditis	[269]
Graves' disease; thyroid adenoma	[269]–[271]
Rheumatoid arthritis; Still's disease	[272]–[277]
Polymyositis	[231], [278]–[283]
Systemic sclerosis	[277], [284], [285]
Systemic lupus erythematosus	[286]
Wegener's granulomatosis; other vasculitides	205; 215 [277], [287]
Anti-phospholipid syndrome	[287]
Cryoglobulinemia	[287]
Ocular toxoplasmosis (retinochorioiditis; uveitis; blurred vision; floaters; macular scars; nystagmus; strabismus; reduced visual acuity; blindness; scleritis; papillitis; retinal necrosis; vasculitis; retinal detachment; vitritis; congenital cataract; neuroretinitis; atrophic optic papilla; retinitis pigmentosa)	[225], [288]–[291]
Glaucoma	[292], [293]
Ovarian dysfunction	[294]–[296]
Uterine atrophy	[297]
Impaired reproductive function (*T. gondii* was present in testicles, epididymis, seminal vesicles, prostate gland in rams, and caused abnormalities in sperm motility, viability and concentration rates, weight of epididymis in rats, orchitis)	[255], [296], [298]–[300]
Nephrotic syndrome; lipoid nephrosis	[250], [251], [255], [301]–[306]
Schönlein-Henoch purpura	[196], [307], [308]
Glomerulonephritis (various forms; including these with development of fibrosis); impaired kidney function	[250], [251], [301], [305], [306], [309]–[312]
Atherosclerosis; obesity; cardiovascular deaths; all-cause mortality	[253], [313]–[318]
Diverse abnormalities in aggregate personality; including aggressive behavior in animals and humans	[9], [319]–[321]

Some of the clinical manifestations of *T. gondii* infection may be as a result of extensive interaction of the pathogen with approximately 3000 host genes or proteins possibly because of frequent host/pathogen antigen homology that disrupts/creates/triggers host specific metabolic pathways, and finally contributes to the development of endophenotypes of different diseases [154]. The parasite and concomitant viral and/or bacterial infections scavenge important metabolites from host cells and/or donate other compounds to the host causing unwanted effects. In addition, *T. gondii*-derived autoantibodies also play an important role in the pathology associated with the parasite [154].

### Association of seroprevalence of toxoplasmosis with other pathologies

Due to the fact that *T. gondii* infection is omnipresent and associated with development of many pathologies in humans and animals, including the disease burden of congenital toxoplasmosis, as represented by disability-adjusted life years (DALY) being the highest among all foodborne pathogens [149], the purpose of this work was to collectively evaluate available geoepidemiological data on the parasite worldwide national seroprevalence variations and their relationship with mortality and disability rates. In the analyses, gross domestic product (GDP) *per capita* as a covariate was used because earlier it was argued and demonstrated that culture-level correlations need to be controlled for regional socioeconomic parameters [9], [215], [322]. If possible (i.e. in multivariate GLM analyses), two potential confounding variables that could strongly influence both the survival of *Toxoplasma* oocysts in soil and a course of various diseases, namely the average latitude (proxy for temperature) and the average relative humidity of particular countries have also been included into the statistical models. In this study we found many positive and some negative associations between the prevalence of toxoplasmosis and various diseases burdens. The number and strength of these associations were much higher than could be expected by chance. Still, it must be emphasized that statistical association does not mean causality. Based on the finding of a statistical association between two phenomena, one cannot determine which of them is the cause and which is the effect – in other words, whether event A causes event B, or whether event B causes event A. Not only we are unable to determine whether event A causes B, or B causes A, but sometimes there is an (unknown) event C that causes both A and B. Therefore, all effects observed in the present exploratory study as well as all suggested biological or medical interpretations must be considered just as potential stimuli for the next, more focused research (search for unknown confounders and for independent evidence) that must follow.

## Methods

### Mortality and Burden of Diseases data

The data on disease burden, mortality and Disability Adjusted Life Year (DALY), were obtained from the table “Mortality and Burden of Diseases Estimates for WHO Member States in 2004” published by WHO [323] and available at: www.who.int/evidence/bod. The publication can be downloaded from the website: http://www.who.int/healthinfo/global_burden_disease/2004_report_update/en/index.html; accessed July 2013). Summary tables present the best estimates of WHO – based on the evidence available in mid-2008 – rather than from the official estimates of Member States. Methods and data sources are summarized in the Annexes of the “Global burden of disease: 2004 update” [323], and the methodology used is described in more details elsewhere [324]; also available at: http://www.dcp2.org/pubs/GBD; accessed July 2013.

The Disability Adjusted Life Year (DALY) has been defined [323] as “a health gap measure that extends the concept of potential years of life lost due to premature death and also to include equivalent years of ‘healthy’ life lost by virtue of being in a state of poor health or disability.” Thus, the DALY combines in one measure the time lived with disability and the time lost due to premature mortality. One DALY can be thought of as one lost year of ‘healthy’ life, while the ‘burden of disease’ as a measure of the gap between current health status and an ideal situation where everyone lives into old age free of disease and disability. The method of calculation of age-standardized DALY has been described earlier [323].

### Data collection for prevalence of toxoplasmosis

In the literature, most of toxoplasmosis prevalence (seroprevalence) data are available only for women in childbearing age. Therefore, all available data collected for this population were published mostly between 1995–2008; the final database was obtained from 88 countries (29 European). When more than one estimation of prevalence of toxoplasmosis was available for a particular country, we gave priority to multicenter studies performed between 1998–2004. When the studies published different prevalence data for various regions or different years we calculated an unweighted arithmetic mean. The obtained data were adjusted to a standard age 22 years to eliminate differences in prevalence caused by different childbearing ages in various countries [325] were kindly provided by Mudhakar Dama) using the formula:
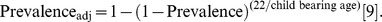



### Statistical Methods

All statistical tests except partial Kendall correlation test were performed independently with SPSS 21 and Statistica 10.0. The association between seroprevalence of toxoplasmosis and specific disease burden estimated with age standardized DALY was calculated using nonparametric partial Kendall correlation test [326], [327] with Gross Domestic Product per capita (GDP) as covariate. Because the results of similar analyses performed with the General Linear Model (GLM) method were qualitatively the same, GLM data were primarily interpreted in the Discussion section of present study. The GLM is more sensitive for the quality of data (e.g. to presence of outliers and non-Gaussian distribution of data, etc.), however, it enables to control for more than one covariate. In the present analysis, we controlled for GDP, geographical latitude and annual mean of relative humidity of particular countries using data available at http://data.worldbank.org/indicator/NY.GDP.PCAP.CD (accessed 10.12. 2013) and http://www.climatemps.com/ (accessed 2.4. 2013). No formal corrections for multiple tests were carried out, however, the fraction of significant results largely exceeded a theoretical value of 5 false positive results per 100 tests.

The medical importance of the association was expressed as regression coefficient ‘B’, the slope of the regression line. The higher is the absolute value of B the greater is the positive or negative impact of the predictor variable (here prevalence of toxoplasmosis) on the dependent variable (here DALY or mortality). The strength of statistical association is expressed as Eta^2^, which reflects the proportion of variance in the dependent variable (the DALY or mortality) associated with or accounted for by each of the main effects, interactions, and error in an ANOVA study (the prevalence of latent toxoplasmosis) [328] pp. 54–55, [329] pp. 317–319. The statistically significant results, i.e. the associations with p value <0.05 and trends, i.e. the associations with p value <0.1 (significant in one-sided but not in two-sided tests) were listed in the tables and in the main text.

## Results and Discussion

### Correlation of toxoplasmosis prevalence with GDP per capita, geolatitude and humidity

**Figure 1:**
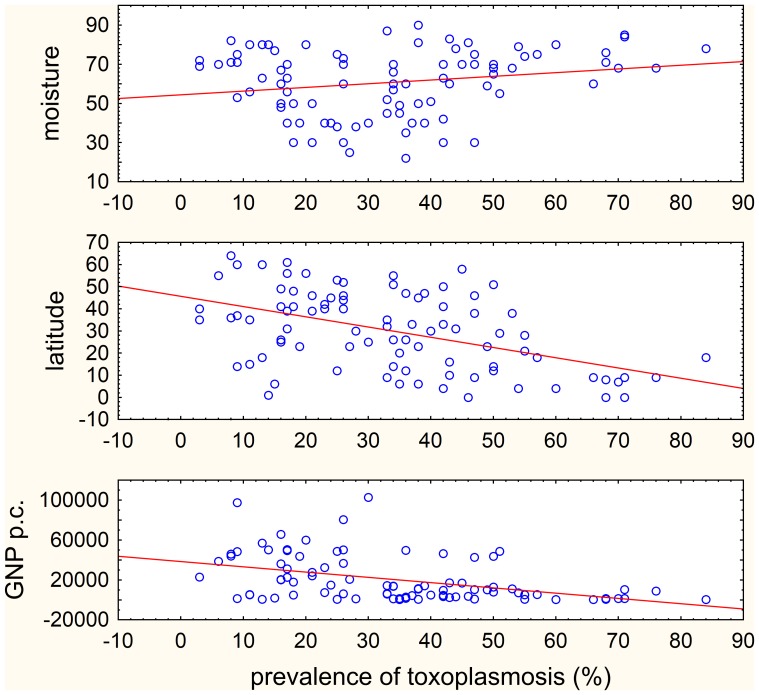
Correlation between prevalence of toxoplasmosis humidity, geolatitude and GDP per capita in all 88 countries. The GDP (1000 $), latitude (°) and relative humidity (%) data are shown only for the region or locality for which latent toxoplasmosis prevalence information (%) is reported.

Fig. 1 suggests that the prevalence of toxoplasmosis correlates with GDP, and possibly also with latitude and humidity for the countries for which the information on toxoplasmosis prevalence is reported. For the whole set of countries (n = 88), the prevalence of toxoplasmosis correlated positively with GDP per capita (Spearman R = −0.484, p<0.001) and latitude (Spearman R = −0.449, p<0.001), and non-significantly positively correlated with the humidity (Spearman R = 0.180, p = 0.093) in the univariate nonparametric Spearman test. For European countries (n = 29) all three correlations were not significant (p>0.151) and for non-European countries (n = 59) the prevalence of toxoplasmosis correlated negatively with GDP per capita (Spearman R = −0.382, p = 0.003) and latitude (Spearman R = −0.396, p<0.002), and positively with humidity (Spearman R = 0.308, p = 0.017). The multivariate GLM analyses with GDP, latitude and humidity as independent variables showed negative correlation with GDP (all countries: p = 0.006, Eta^2^ = 0.085; European: p = 0.044, Eta^2^ = 0.153; non-European: p = 0.022, Eta^2^ = 0.092). For the latitude and humidity, the results differed between European and non-European countries (latitude – all countries: p = 0.014, Eta^2^ = 0.070; European: p = 0.055, Eta^2^ = 0.135; non-European: p = 0.073, Eta^2^ = 0.057; humidity – all countries: p = 0.056, Eta^2^ = 0.043; European: p = 0.037, Eta^2^ = 0.162; non-European: p = 0.356, Eta^2^ = 0.016). These results suggest that the GDP, and possibly also the latitude and humidity, should be incorporated into the statistical models as covariates.

### Correlation of toxoplasmosis prevalence with age-standardized DALY for diseases

**Figure 2:**
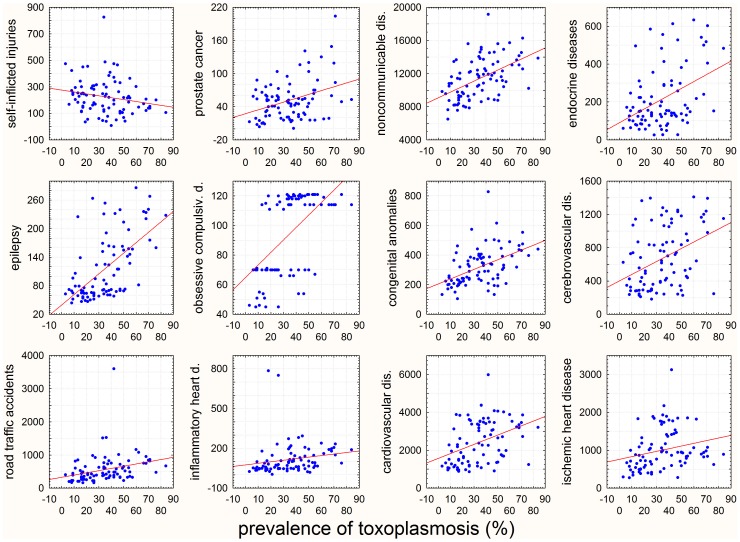
Correlation of prevalence of toxoplasmosis with various disease-attributed DALY for 88 WHO-member countries. The x-axes show prevalence of toxoplasmosis (%) in women of childbearing age and y-axes the number years of ‘healthy’ life lost by virtue of being in a state of poor health or disability due to particular disease per 100,000 inhabitants in 2004.

The present study showed that prevalence of toxoplasmosis correlated with specific disease burden measured with age-standardized DALY or with specific mortality in particular countries (Fig. 2).

Because distribution of DALY and mortality for many diseases was not normal, the analysis of association of toxoplasmosis prevalence with disease burden was performed with two methods, nonparametric partial Kendall correlation test and GLM analysis.

**Figure 3:**
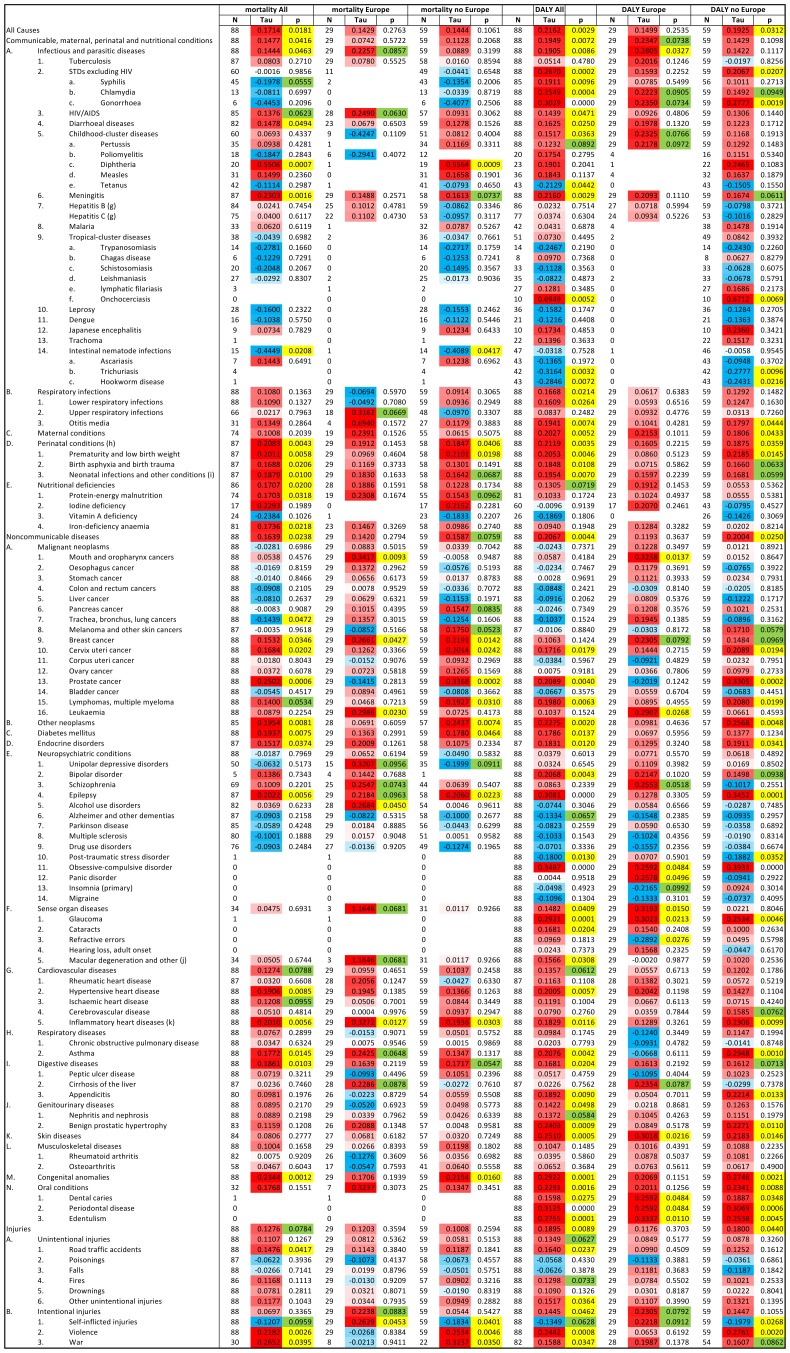
Correlation of mortality and Disability Adjusted Life Year (DALY) with prevalence of toxoplasmosis for all 88 WHO member countries (29 European and 59 non-European countries). The correlations were estimated with partial Kendall correlation test with GDP per capita as covariate. Positive Kendall Taus (red) correspond to positive and negative Taus (blue) to negative correlations. Significant results (p<0.05) are labeled with yellow and trends (p<0.10) with green colors.

Since, a nonparametric partial Kendall correlation test enables to control for one confounding variable, we controlled for the GDP per capita because this variable is known to be strongly correlated with the quality of health care and therefore with the burden associated with many diseases. The partial Kendall correlation test demonstrated that age standardized DALY of 57 of 128 diseases and disease categories on the WHO list showed significant correlation (53 positive and 4 negative) with prevalence of toxoplasmosis in all (n = 88) countries after the effect of GDP was controlled, and further 8 diseases showed such trends (p<0.1) (6 positive and 2), see Fig. 3. Similar analyses for 29 European countries showed 12 significant correlations (11 positive and 1 negative) and 11 trends (10 positive and 1 negative), and for 59 non-European countries test revealed 33 significant correlations (29 positive and 4 negative) and 10 trends (all positive), Fig 3.

**Figure 4:**
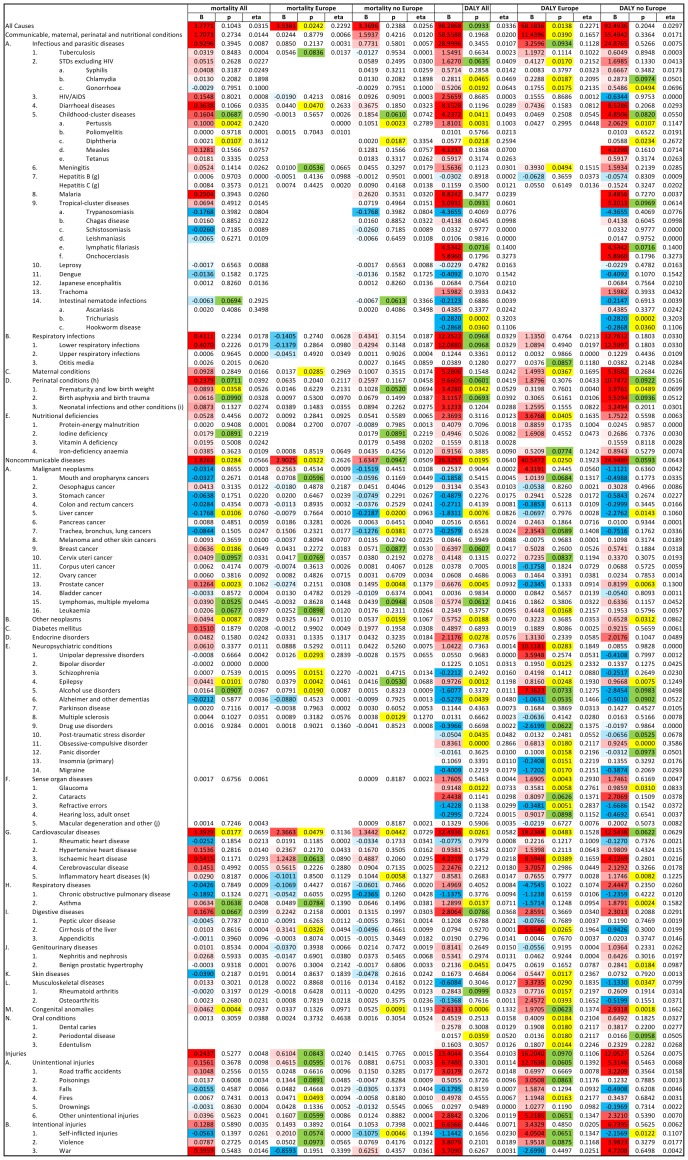
Correlation of mortality and Disability Adjusted Life Year (DALY) with prevalence of toxoplasmosis for all 88 WHO member countries (29 European and 59 non-European countries). The correlations were estimated with General Linear Model with GDP per capita, latitude humidity, as covariates. Positive B (red) correspond to positive, and negative B (blue) to negative correlations. Significant results (p<0.05) are labeled with yellow and trends (p<0.10) with green colors.

GLM analyses with GDP, latitude and humidity as covariates showed that age standardized DALY of 23 of 128 diseases and disease categories on the WHO list had significant correlation (18 positive and 5 negative) with prevalence of toxoplasmosis in all (n = 88) countries after the effect of GDP was controlled, and further 12 diseases showed such trends (p<0.1) (all positive), Similar analyses for 29 European countries showed 32 significant correlations (29 positive and 3 negative) and 18 trends (16 positive and 2 negative), and for 59 non-European countries had 18 significant correlations (13 positive and 5 negative) and 13 trends (9 positive and 4 negative), Fig 4.

### Correlation of toxoplasmosis prevalence with disease mortality

Partial Kendal correlation tests also showed that mortalities from 31 of 111 diseases and disease (WHO) categories with nonzero mortality had significant correlation (29 positive and 2 negative) with prevalence of toxoplasmosis in 88 countries after the effect of GNP was controlled, and further 6 diseases showed such trends (p<0.1) (5 positive and 2 negative), see Fig. 3. Similar analyses performed for 29 European countries demonstrated 6 significant correlations (all positive), and 11 trends (all positive) (here only 90 diseases had the mortality data necessary for analysis), and for 59 non-European countries showed 16 significant correlations (14 positive and 2 negative) and 8 trends (7 positive and 1 negative) (109 diseases had enough data for the analysis) (Fig. 3).

GLM analyses also showed that mortalities of 12 out of 111 diseases and disease categories (for 17 diseases the mortality data were available for less than 3 countries) revealed significant correlation (11 positive and 1 negative) with prevalence of toxoplasmosis in 88 countries after the effects of GDP, latitude humidity were controlled, and further 11 diseases showed such trends (10 positive and 1 negative), see Fig. 4. Similar analyses performed for 29 European countries showed 11 significant correlations (all positive) and 13 trends (all positive), and for 59 non-European countries had 11 significant correlations (8 positive and 3 negative) and 8 trends (7 positive and 1 negative), Fig. 4.

Several explanations may be put forward for positive correlation between prevalence of latent toxoplasmosis and the DALY or the morbidity from a particular disease: a) *T. gondii* infection may increase the risk of development of some diseases, b) certain diseases may increase the risk of acquiring toxoplasmosis, or c) some unknown factor(s) may increase both the risk of triggering certain diseases and *Toxoplasma* infection. Similarly, there are several possible explanations for the observed negative correlations: a) infection with the parasite can increase resistance/tolerance of the infected host to a certain disease by modulating its innate and/or acquired cellular/humoral immunity. For example, suppression of cellular immunity observed in *in vivo* as well as *in vitro* systems can make the host more sensitive to infection by certain pathogens, and at the same time protect it against development of some autoimmune diseases. On the other hand, chronic inflammation loci in tissues/organs can be responsible for inducing health problems, including development of certain tumors [214], [215], and at the same time activation of the host immune system can make local tissue environment unfavorable for growing, proliferation and persistence of certain infectious agents. For example, toxoplasmosis increases dopamine levels in the brain tissue, which can protect the host against symptoms of certain diseases, e.g. Parkinson's disease, and at the same time it can increase the risk for development of other pathologies, such as schizophrenia [330], [331].

In the present GLM analyses, three potential confounding factors, the GDP *per capita* (which strongly correlates with quality of health services and hygienic standards), the geolatitude (which strongly correlates with temperature and quantity and quality of sunlight) and humidity which influences survival of *Toxoplasma* oocysts in soil), were controlled. However, many other factors, such as cultural habits, can also influence the risk of *Toxoplasma* infection, and/or other infections as well. It has been, for example, suggested that toxoplasmosis could be a sexually transmitted disease (STD) transferred from men to women with semen/ejaculate [331], [332]. This could explain the observed positive correlation between the prevalence of latent toxoplasmosis and DALY for several STDs. Finally, it is to be noted that the incidence of a certain disease can be decreased by an increased risk of death due to another concomitant disease.

In the next part of discussion, comments are made on the results obtained for particular diseases, mainly the results of GLM tests as the partial Kendall correlation tests can control for one confounding variable only and the regression coefficient (B-value) has more straightforward interpretation than Kendall Tau. We have concentrated on the age-standardized DALY data because only a subset of disease could result in the death of patients under normal conditions. The strength of correlation is usually estimated by Eta^2^, which reflects fraction of variability of dependent variable that can be explained by an independent variable (here, by the prevalence of toxoplasmosis). The clinical relevance of a particular association is however better reflected by the regression coefficient B, which shows an increase of a dependent variable (here, the age standardized DALY expressed in years of life lost due to premature death per 100 000 inhabitants) that corresponds to the increase of an independent variable per one unit (here, the increase of prevalence of toxoplasmosis by 1%). Therefore, the B-value reflects not only the strength of the correlation but also the incidence of particular disease.

### Association of seroprevalence of toxoplasmosis with specific diseases in all 88 countries

#### All disease burden

Prevalence of toxoplasmosis explained about 23% of between-countries variability in mortality and age-standardized DALY in Europe (mortality: B = 3.538, Eta^2^ = 0.229, p = 0.024; DALY: B = 68.18, Eta^2^ = 0.227, p = 0.014). This association was not significant for non-European countries (mortality: B = 3.37, Eta^2^ = 0.026, p = 0.239; DALY: B = 92.49, Eta^2^ = 0.030, p = 0.204) or for all 88 countries (mortality: B = 3.78, Eta^2^ = 0.031, p = 0.104; DALY: B = 98.287, Eta^2^ = 0.034, p = 0.093). Both communicable and noncommunicable diseases were responsible for the observed association in Europe, however, for communicative infection was significant only for DALY (mortality: B = 0.024, Eta^2^ = 0.007, p = 0.878; DALY: B = 11.44, Eta^2^ = 0.166, p = 0.039).

#### Noncommunicable diseases

The highest regression coefficient (B = 26.33, p = 0.019) was found for the entire category of noncommunicable diseases. In this case, a difference of 1% in the prevalence of toxoplasmosis corresponded to a difference of 26.33 DALY per 100,000 inhabitants. The prevalence of toxoplasmosis explained 6.4% of between countries variability in DALY.

#### Cardiovascular diseases

The second highest regression coefficient (B = 12.49, p = 0.026, Eta^2^ = 0.058) was observed for cardiovascular diseases. However, prevalence of toxoplasmosis explained about 15% of variability in mortality attributed to cardiovascular diseases in European countries subset (B = 18.23, p = 0.048, Eta^2^ = 0.153). Also, in the European countries, the difference in prevalence of toxoplasmosis explained about 17% of variability of ischemic heart disease (B = 8.59, p = 0.039, Eta^2^ = 0.166). Stronger correlations between prevalence of toxoplasmosis and heart disease, especially inflammatory heart disease, were revealed with nonparametric partial Kendall test (comparing the data in tables shown in Fig. 3 and 4). One may suggest that the non-Gaussian distributions of dependent variables, e.g. a bimodal distribution for cerebrovascular and cardiovascular disease and highly skewed distribution for hypertensive, rheumatic and inflammatory heart diseases (results not shown), were responsible for the false negative results of the GLM tests.

Theoretically, the inability to control for more than one confounding variable (here, the latitude and annual precipitation) in the distribution-robust nonparametric tests could be responsible for the false positive results of the partial Kendall test. However, the present data do not support this explanation. Fig. 1 shows that the latitude correlates negatively and the humidity positively with the prevalence of toxoplasmosis. The Kendall correlation test showed, for example, the positive association between hypertensive heart disease and prevalence of toxoplasmosis (DALY: p = 0.06; mortality: p = 0.008), while GLM demonstrated lack of such association (DALY: p = 0.345; mortality: p = 0.282). The negative association between prevalence of toxoplasmosis and latitude (see above) could explain the positive correlation between the disease burden for hypertensive heart disease and prevalence of toxoplasmosis, because the disease burden for hypertensive heart disease correlated negatively with the latitude (DALY: B = −3.846, Eta^2^ = 0.139, p<0.001), but also negatively with humidity (B = −2.145, Eta^2^ = 0.059, p = 0.024), which is in contradiction to the explanation). However, the negative correlation between prevalence of toxoplasmosis and latitude could not explain the positive correlation between prevalence of toxoplasmosis and the inflammatory heart disease (DALY: p = 0.012, mortality: p = 0.006), because inflammatory heart disease correlated positively with latitude (B = 0.749, Eta^2^ = 0.010, p = 0.362), and negatively with humidity (B = −1.392, Eta^2^ = 0.041, p = 0.060). This contradicts the notion that the correlations with the latitude or humidity could be responsible for the association between toxoplasmosis and cardiovascular diseases detected with the partial Kendall correlation tests.

#### Perinatal conditions

The third highest regression coefficient (B = 9.66) was demonstrated for this category, but, it explained only 3.3% of the variability making the correlation non-significant (p = 0.097). The important components of this category were prematurity and low birth weight (B = 3.43; Eta^2^ = 0.053; p = 0.034). Published data suggest that early development of embryos in mothers with latent toxoplasmosis was slower, although, the birth weight of newborns was approximately the same as those of infection-free mothers [26]. These studies were performed in Czech Republic, a developed European country, with low frequency of virulent *T. gondii* strains. It is possible that the effect of toxoplasmosis on development of embryos is qualitatively different in other parts of the world. It is indicative that the association between prevalence of toxoplasmosis and DALY for prematurity and low birth weight is much weaker for the European countries (B = 0.320; Eta^2^ = 0.004; p = 0.761) than for others. Once again, the correlation of DALY for perinatal conditions with prevalence of toxoplasmosis detected with nonparametric tests were stronger than those detected with GLM. Here, however, the correlation observed in Kendall test (in which the GDP but not the latitude and humidity was controlled) can be explained by correlation of both prevalence of toxoplasmosis and the DALY for perinatal conditions, controlling for the latitude, because the latter correlation is negative and relatively strong (and significant).

#### Congenital abnormalities

The regression coefficient was only medium (B = 2.613; Eta^2^ = 0.133; p<0.001). The correlation was significant for the 88 countries, however, it was non-significant for the European countries (B = 1.970; Eta^2^ = 0.137; p = 0.062). Interestingly, more than 55 years ago, it was observed that children with Down syndrome had a much higher probability of having mothers with latent toxoplasmosis (84%) than normal children (32%) [333]; the probability of having fathers with latent toxoplasmosis did not differ between children with and without this disorder. Recently, it has been suggested that Down syndrome may be caused by congenital *T. gondii* infection [182], Table 2. This hypothesis is supported by the finding that *T. gondii* has a specific protein transporter exposed at the parasite surface, with high affinity for folic acid, which is responsible for the acquisition and salvaging of exogenous folate compounds [187], thus leading to folate deficiency in the host. The transport of folic acid across the parasite plasma membrane was found to be rapid, biphasic, bidirectional, specific, and concentration- and temperature-dependent, and methotrexate, an antifolate, was found to be internalized by the protozoan pathogen to the mitochondrion [187]. In addition, it has been demonstrated that simultaneous dietary restriction of folic acid and infection with *T. gondii* induces DNA damage in peripheral blood cells of infected mice [186]. Furthermore, *T. gondii* infection was also associated with nutritional deficiencies of iron and iodine [266], [334], [335], which may lead to have adverse effect on the growth and development of the fetus.

Principally different explanation of the observed association suggest results of three studies on the influence of toxoplasmosis on secondary sex ratio and on the rate of prenatal and early postnatal development of children of infected mothers, These results indicate that latent toxoplasmosis could protect the embryos with less serious developmental disturbances against spontaneous abortion [26], [336]–[338]. It is possible that such beneficial activity of the parasite could translate into positive correlation between prevalence of toxoplasmosis and incidence and severity of congenital abnormalities.

#### Lymphatic filariasis

The regression coefficient was medium (B = 4.534; Eta^2^ = 0.140, p = 0.072). This disease occurs in 27 countries of our data set and therefore the highly non-Gaussian distribution of DALY (and mortalities) makes the results of GLM analysis not fully credible. The nonparametric test showed no significant association between filariasis and toxoplasmosis. However, possible relationship between toxoplasmosis and filariasis could theoretically be explained by the fact that *T. gondii* usually disseminates via lymphatic system in the infected patients, who usually have symptomatic lymphadenopathy. In addition, possible interactions exist between toxoplasmosis-associated changes in the host lymphatic system and a progressive clinical picture of lymphatic filariasis (Table 2). Filariasis may therefore represent another co-morbidity of the host infected with *T. gondii*.

#### Measles

The regression coefficient was medium (B = 4.124; Eta^2^ = 0.070; p = 0.137), and the correlation was not significant. A possible association, if it really exists, is difficult to rationalize, however, latent cerebral toxoplasmosis could influence susceptibility to measles because of changes in the immune status of the children (Table 2) [339], [340] caused by the parasite or measles-mumps-rubella (MMR) vaccination.

#### Asthma

The regression coefficient B was 1.290 (Eta^2^ = 0.071, p = 0.014). An opposite direction association was observed for European (B = −1.571, Eta^2^ = 0.096, p = 0.124) and non-European (B = 1.879, Eta^2^ = 0.158, p = 0.002) countries. We have no explanation for the positive association, but, the negative association between *T. gondii* infection and asthma could be, at least partially, explained by the anti-inflammatory effect of histamine produced in excess in asthmatic patients, since, asthma is a chronic inflammatory disorder associated with an increased number of T_H_2 (T helper type 2) cells producing anti-inflammatory cytokines and decreased number of T_H_1 (T helper type 1) cells generating pro-inflammatory cytokines. Histamine modulates the cytokine T_H_1/T_H_2 balance because it enhances secretion of T_H_2 cytokines, such as IL-4, IL-5, IL-10, and IL-13, and inhibits production of T_H_1 interleukins (IL-2, IFN-γ, and monokine IL-12) [341], thus exerting beneficial anti-inflammatory effects.

#### Epilepsy

Epilepsy had a small regression coefficient (B = 0.972; Eta^2^ = 0.112, p = 0.001), but the correlation was highly significant. This association was observed both in European (B = 0.816, Eta^2^ = 0.193, p = 0.025) and non-European countries (B = 0.967, Eta^2^ = 0.125, p = 0.007). The association between latent toxoplasmosis and cryptic epilepsy has already been suggested to exist on the basis of the case control studies – for example, see Ref. [342], [343] Table 2.

#### Leukemia

Surprisingly, there was a strong association between toxoplasmosis and DALY for leukemia in European countries (B = 0.445, Eta^2^ = 0.216, p = 0.017) explaining about 22% of variability in DALY. In a small study performed in 15 patients with leukemia, 10 (66.7%) individuals had increased serum IgG, and 2 also had increased IgM antibodies to *T. gondii* [219]. It is known that tachyzoites of *T. gondii* use a “Trojan horse” strategy to penetrate various tissues and organs of the infected host. They even transform the phenotype of infected white cells by, for example, increasing migratory activity of the infected dendritic cells [344] and by inhibiting apoptotic activity of the infected cells [345]–[349]. It is also possible that the increased risk of various forms of cancer, including leukemia, could be as a result of infection with *T. gondii*, which may cause a nonspecific chronic local inflammation.

#### Cancer of the mouth/oropharynx

The regression coefficient was small (B = 1.014; Eta^2^ = 0.132, p = 0.067) and the correlation was non-significant (positive for European but negative for non-European countries). A typical symptom of acute toxoplasmosis is tonsillitis. Thus, it is possible that tonsillitis leading to the development of local chronic inflammation may result in inducing precancerous changes in predisposed individuals. Association of prevalence of toxoplasmosis with cancer of the larynx in men and women, and lung cancer in men (but not with cancer of oropharynx), has been reported [214]. In addition, one cannot exclude that frequent oral sex could, at least in part, affect this correlation, since, the parasite has been found in the semen and ejaculate of both animals and humans infected with *T. gondii* [331], [332], see also Table 2.

#### Prostate cancer

Prostate cancer had a B of 0.667 (Eta^2^ = 0.093, p = 0.005). An association in opposite direction was observed for European (B = −0.235, Eta^2^ = 0.091, p = 0.133) and non-European (B = 0.820, Eta^2^ = 0.130, p = 0.006) countries. Benign prostate hypertrophy had a B of 0.214, (Eta^2^ = 0.482, p = 0.045) and this association was positive but non-significant for non-European countries (B = 0.062, Eta^2^ = 0.079, p = 0.165) and positive and significant (B = 0.284, Eta^2^ = 0.098, p = 0.018) for European countries. It is possible that the increased incidence of prostate cancer and hypertrophy could be related to the increased concentration of testosterone as observed in *Toxoplasma*-infected male rats [350] and men [351], [352]. Histopathologic studies of the reproductive system in male sheep experimentally infected with the parasite showed inflammatory process in the prostate gland and seminal vesicles strongly suggestive of *Toxoplasma* infection [300] (Table 2). Thus, persistent chronic inflammation caused by the parasite also must be taken into account in the development of prostate cancer, although perhaps having different clinical courses in European versus non-European countries.

#### Obsessive compulsive disorder

Obsessive compulsive disorder (OCD) had a B value of 0.836, but the prevalence of toxoplasmosis explained 28.7% of total variability in DALY (p<0.001). For European countries the association was weaker (B = 0.681, Eta^2^ = 0.212, p = 0.018), but for non-European countries, it was nearly two times higher (B = 0.925, Eta^2^ = 0.358, p<0.001). The association between latent toxoplasmosis and OCD has already been suggested to exist on the basis of results of a case-control study [176], in which the authors found 47.6% seroprevalence of toxoplasmosis among OCD patients (n = 42) and only 19% prevalence in controls (n = 100). Both neurotransmitters, dopamine and serotonin, are expected to play an important role in OCD. It is possible that either an increased concentration of dopamine, synthesized by two enzymes encoded in *T. gondii* genome, and a decreased level of serotonin, the metabolite of tryptophan degradation – the part of the host defense against parasitic infection – could be important etiologic factors in the development of OCD. Incidentally, OCD is not an uncommon disease (incidence of about 3%) and is probably associated with an increased risk of suicide [353]–[356]. It can be speculated that an association between toxoplasmosis and OCD could, in part, be responsible for the increased risk of suicides reported in *Toxoplasma*-infected individuals. It is possible that the nearly twice stronger relationship between toxoplasmosis and OCD in European (Eta^2^ = 0.202) than in non-European (Eta^2^ = 0.360) countries could somehow be related with the negative, not positive, relationship between the prevalence of toxoplasmosis and incidence of suicides in non-European countries observed in our study, see below.

#### Endocrine disorders

A regression coefficient (B) of 2.118 was observed for the category of ‘endocrine disorders’. The prevalence of toxoplasmosis explained about 5.8% of total variability (p = 0.028) and non-significant trends were observed for both European and non-European countries. The positive and negative associations between toxoplasmosis and testosterone concentration were observed for men and women, respectively. However, our unpublished data suggest that toxoplasmosis could also play a role in the production of thyroid hormones. This finding is supported by recent literature data demonstrating the prevalence of anti-*Toxoplasma* IgG antibodies in patients with thyroid autoimmunity [277], [357] as well as in many other autoimmune diseases, as compared with controls (Table 2). The autoantibody burden has also been demonstrated even in non-autoimmune individuals during infections [358]. Patients with autoimmune diseases frequently present neurologic manifestations [359], and this may further support the significant prevalence of toxoplasmosis in patients with endocrine disorders, because central nervous system is the most immunoprivileged organ for *T. gondii* dissemination and settlement in the host. Fetal [360] and maternal microchimerism, acting as a “Trojan horse” in dissemination of the parasite, could play an important role in endocrine and other health disorders [361].

#### Sexually transmitted diseases (STDs)

A regression coefficient (B) of 1.63 was observed for the general category of ‘sexually transmitted diseases except AIDS’. Prevalence of toxoplasmosis explained 4.1% of variability (p = 0.063). The association was stronger in European countries (B = 0.413, Eta^2^ = 0.215, p = 0.017) than in non-European countries (B = 1.699, Eta^2^ = 0.041, p = 0.133). Similar effects were observed for gonorrhea (B = 0.175, Eta^2^ = 0.213, p = 0.018) and chlamydia (B = 0.229, Eta^2^ = 0.209, p = 0.019) in Europe, but were different for non-European countries for gonorrhoea (B = 0.549, Eta^2^ = 0.069, p = 0.049) and chlamydia (B = 0.287, Eta^2^ = 0.050, p = 0.097). We believe that the correlation of both prevalence of toxoplasmosis and STD with other factor(s), such as a risky sexual behavior (promiscuity and frequent unprotected sex) is responsible for the observed positive association between prevalence of toxoplasmosis and age/controlled DALY for STDs. It has been suggested by other investigators [332] that *Toxoplasma* is frequently transmitted by ejaculate in several animal species. An indirect evidence exists that the same could also occur in humans [331]. Practicing oral sex or even kissing may also be another important route of wide dissemination of the parasite among sexual partners, in addition to intercourse.

#### Pertussis

Pertussis had a regression coefficient (B) of 1.81. Prevalence of toxoplasmosis explained 10% of variability in DALY (p = 0.003). This correlation could, at least in part, be rationalized by the findings that in some instances immunization could shorten the incubation period of certain diseases or convert a latent infection/inflammation into clinically active disease. The necessary precondition for such an occurrence is the presence of latent infection or asymptomatic bacterial/viral/parasitic colonization [362].

It has been reported that some infants and young children develop various urinary tract diseases, such as acute renal failure, nephrotic syndrome, or pyelonephritis, after the injection of the whole-cell DTP vaccine [363]. Administration of DTP vaccine caused dose-, and time-dependent biological changes in animals, including increased hepatic mRNA expression for several cytokines, marked inhibition of liver CYP450 enzymes activity, induction of IFN-γ, and enhanced NOS mRNA expression [364], [365]. In addition, a significant increase in toxoplasma-cysts was observed in brain tissues of mice exposed to both *T. gondii* infection and methylmercury (thimerosal, a vaccine preservative) versus the parasite alone [366]. Thus, it seems that a concomitant use of strong lipopolysaccharide antigen (a component of the whole-cell pertussis) and thimerosal exerted serious synergistic adverse health effects when given to individuals with latent central nervous system *T. gondii* infection [184], [340]. It is not clear, however, whether the incidence of pertussis correlates with intensity of DPT vaccination.

#### Childhood cluster diseases

The regression coefficient was high (B = 4.24), and it explained 4.9% of variability (p = 0.041). The high regression coefficient is not surprising because, for example, respiratory tract diseases are the most frequent cause of hospitalization and death in children and *T. gondii* infection is extensively prevalent worldwide [148], [367].

There are many predisposing, provocative, facilitating, and other factors, such as chronic hypoxia, viral infections/bacterial toxins, inflammatory states, biochemical disorders, and genetic abnormalities that are the most likely triggers of development of respiratory tract diseases, including sudden infant death syndrome [368]. For example, exposure of children to cigarette smoke (second-hand) increased their susceptibility to viral and bacterial infections because children who died of sudden infant death syndrome had markedly higher concentration of cotinine (a metabolite of nicotine) in their lung tissue and pericardial fluid than in controls [369], [370]. Thus, negative effects of latent toxoplasmosis on children's physiology, including function of the immune system, may markedly affect clinical course of diseases in children and might significantly affect disease severity and mortality.

#### Suicides

Latent toxoplasmosis was found to be associated with an increased risk of attempted suicides [161], [170], [171], [371]. The results of earlier correlation studies performed for 17–20 European countries [172], [173] were confirmed by the present study, wherein a positive trend was observed for the 29 European countries (B = 4.05, p = 0.065, Eta^2^ = 0.135), but, this correlation could not be detected for the entire 88 countries. In fact, a negative correlation between risk of suicide and prevalence of latent toxoplasmosis was found for the 59 non-European countries (B = −2.157, p = 0.012, Eta^2^ = 0.11). We have no explanation for this qualitative difference between European and non-European countries. However, the existence of positive correlation between the prevalence of latent toxoplasmosis and violence [174] was confirmed by partial Kendall correlation for all countries (Tau = 0.244, p = 0.001) and non-European countries (Tau = 0.276, p = 0.002), but not for European countries (Tau = 0.065, p = 0.619), the GLM method showed just a trend for European countries (see tables shown in Fig. 3 and Fig. 4).

#### Traffic accidents

On the basis of four published case-control studies, a positive correlation was expected between the burden of traffic accidents and prevalence of latent toxoplasmosis. However, this correlation was significant in nonparametric tests (mortality: Tau = 0.148, p = 0.042; DALY: Tau = 0.164, p = 0.023). Weak correlation in other tests may be caused by the fact that the traffic accident rates depend on many confounding variables, including the number of vehicles in circulation, length of the road network, mean number of kilometers (miles) travelled by one inhabitant per year, driver behavior (alcohol/drug use, sleep deprivation, etc.), etc. Probably, much stronger correlations would be detected when these variables would be included into the models. Interestingly, the strongest association of latent toxoplasmosis and traffic accidents was found for RhD negative drivers [372], while the RhD positive subjects, especially RhD positive heterozygotes seem to be relatively protected against impairment of reaction times [373], [374] as well as against traffic accidents [372] (the RhD refers to “Rhesus factor” with immunogenic D antigen, while RhAG indicates no Rh antigens on red blood cell membranes). The lack or deficiency of RhAG proteins in the host red blood cell membrane and an impaired function of aquaporin P1 and P4 water/gas channels in the central nervous system could be associated with various degrees of brain hypoxia [339], thus affecting usual driving performance possibly in synergy with the effects of toxoplasmosis on reaction times and ability of long-term concentration [373]–[375]. Since RhD negative individuals are rare in African and Asian populations [376], an association between traffic accidents and prevalence of toxoplasmosis can be expected mainly in countries inhabited by Caucasians.

### Limitations of the study

It is possible that some of the toxoplasmosis prevalence data are inaccurate, as there are no published national survey data of latent toxoplasmosis carried out systematically. In addition, surveys performed in a relatively small, and ethnically and sociologically homogeneous population, such as in the Czech Republic, demonstrate that seroprevalence of toxoplasmosis varies considerably in different regions of the country. Therefore, it is difficult to estimate the average prevalence of this clinical entity in women of child-bearing age in a particular country on the basis of one or two studies performed in one hospital or even in one city. Furthermore, it is important to point out that the different serological methods used to obtain toxoplasmosis seroprevalence data are not standardized, and vary in sensitivity, specificity, and predictive values. As a consequence, no two tests produce the same results in all cases, even when carried out in the same laboratory [5].

For the present study, probably most of available data for the period 1995–2008 were collected, and published information for period prior to 1995 was also considered. To maximally avoid possible subjective bias, we completed our data set on January 2013 and did not change it after starting the analyses despite of the fact that data for other four countries appeared during 2013. To further decrease the risk of subjectivity in selection of countries, we included available data for all countries; our data set contained the prevalence of toxoplasmosis in 88 countries, which represented the largest ever data set analyzed in all toxoplasmosis correlation studies. To increase the reliability of our results, we confirmed the results of parametric GLM analysis with the nonparametric Kendall test, which is less sensitive to contamination of data with few incorrect values. It may be noted that lack of precision in the prevalence data increases the risk of false negative but not false positive results of correlation.

The existence of a factor correlating with both the prevalence of latent toxoplasmosis and the disease burden can lead to a false positive value in correlation studies. We controlled for one potential confounding variable (GDP) in partial Kendall test and for three potential confounding variables (GDP, latitude and humidity) in GLM tests. It is possible that some unknown factor(s), such as hygienic or eating habits, could influence both the prevalence of latent toxoplasmosis and incidence or morbidity of certain diseases. Existence of such factor(s) could be revealed by confirming present analyses with another set of countries. In the present study, data from all 88 countries were analyzed, and also separately for the European and non-European countries. It is important to repeat the correlation studies based upon independent data sets (if available) for particular regions (such as in France) or various states (such as in USA).

It is quite probable that the incidence of particular diseases reflects better the prevalence of latent toxoplasmosis in an unknown past, rather than the present prevalence. In many countries, the prevalence of latent toxoplasmosis in young women is changing: in some cases it is increasing (China, Korea and Mexico) and in some it is decreasing (most of European countries and USA). The prevalence of latent toxoplasmosis in a general population (the parameter which probably better correlates with disease burden) is more stable because it reflects past rather than present epidemiological situation in particular countries. Still, our lack of knowledge of optimal interval between toxoplasmosis survey and disease burden surveys increases the risk of false negative results of the obtained correlation studies.

## Conclusions

The present results suggest that the prevalence of latent toxoplasmosis in particular countries correlated (mostly positively) with various disease burden measured with age standardized Disability Adjusted Life Years or with age standardized mortality. It must be emphasized that no epidemiological study and especially no correlation (ecological) study can prove existence of causal relation between the two factors. At the same time, results of such studies could indicate which hypothesis should be tested in the future. It is highly probable that some of the observed correlations represent “false correlations” – either the Type 1 errors of used statistical tests or the expression of existence of unknown factor(s) that correlates with both the risk of latent toxoplasmosis and incidence (or severity) of particular disease. However, it is also highly probable that at least some of the observed correlations do occur because toxoplasmosis is, up to now rarely suspected, etiological agent of particular diseases. Existence of some correlations could be expected to happen on the basis of our present knowledge for certain diseases (for example, epilepsy, obsessive compulsive disorder, congenital abnormalities). Some of the obtained correlations may be regarded as rather surprising and should therefore be studied in more detail in the future. In the opinion of the authors, slowly emerging important role of latent toxoplasmosis in etiology of several clinical entities deserves much more attention and financial support in future clinical research.

## References

[1] Nicolle C, Manceaux L (1908) Sur une infection à corps de Leishman (ou organismes voisins) du gondi. C R Acad Sci (Paris) 147: 763–766.

[2] Furtado JM, Smith JR, Belfort R Jr, Gattey D, Winthrop KL (2011) Toxoplasmosis: a global threat. J Glob Infect Dis 3: 281–284. 21887062 10.4103/0974-777X.83536 PMC3162817

[3] Dubey JP, Beattie CP (1988) Toxoplasmosis of animals and man. Boca Raton, Fla.: CRC Press. pp. 1–220.

[4] Dubey JP (1998) Advances in the life cycle of *Toxoplasma gondii* . Int J Parasitol 28: 1019–1024. 9724872 10.1016/s0020-7519(98)00023-x

[5] Tenter AM, Heckeroth AR, Weiss LM (2000) *Toxoplasma gondii*: from animals to humans. Int J Parasitol 30: 1217–1258. 11113252 10.1016/s0020-7519(00)00124-7 PMC3109627

[6] Jones JL, Kruszon-Moran D, Wilson M, McQuillan G, Navin T, et al (2001) *Toxoplasma gondii* infection in the United States: Seroprevalence and risk factors. Am J Epidemiol 154: 357–365. 11495859 10.1093/aje/154.4.357

[7] Scallan E, Hoekstra RM, Angulo FJ, Tauxe RV, Widdowson MA, et al (2011) Foodborne illness acquired in the United States-major pathogens. Emerg Infect Dis 17: 7–15. 21192848 10.3201/eid1701.P11101 PMC3375761

[8] Pawłowski Z (1994) Toxoplasmosis. In: Januszkiewicz J, editor. The outline of infectious diseases in the clinic. 2nd ed. Warsaw: PZWL. pp. 214–218.

[9] Lafferty KD (2006) Can the common brain parasite, *Toxoplasma gondii*, influence human culture? Proc R Soc Biol Sci Ser B 273: 2749–2755. 10.1098/rspb.2006.3641 PMC1635495 17015323

[10] Maggi P, Volpe A, Carito V, Schinaia N, Bino S, et al (2009) Surveillance of toxoplasmosis in pregnant women in Albania. New Microbiol 32: 89–92. 19382673

[11] Rickard E, Costagliola M, Outen E, Cicero M, Garcia G, et al. (1999) Toxoplasmosis antibody prevalence in pregnancy in Buenos Aires Province, Argentina. Clin Microbiol Infec 5: 171–1721.

[12] Karunajeewa H, Siebert D, Hammond R, Garland S, Kelly H (2001) Seroprevalence of *varicella zoster* virus, parvovirus B19 and *Toxoplasma gondii* in a Melbourne obstetric population: implications for management. Aust N Z J Obstet Gynaecol 41: 23–28. 11284642 10.1111/j.1479-828x.2001.tb01289.x

[13] Moese JR, Vander-Moese A (1998) Mother-child pass in Austria and primary toxoplasmosis infections in pregnant women. Cent Eur J Public Health 6: 261–264. 9919373

[14] Tabbara KS, Saleh F (2005) Serodiagnosis of toxoplasmosis in Bahrain. Saudi Med J 26: 1383–1387. 16155652

[15] Ashrafunnessa, Shahla K, Islam MN, Huq T (1998) Seroprevalence of *Toxoplasma* antibodies among the antenatal population in Bangladesh. J Obstet Gynaecol Res 24.. 10.1111/j.1447-0756.1998.tb00061.x 9631599

[16] Breugelmans M, Naessens A, Foulon W (2004) Prevention of toxoplasmosis during pregnancy—an epidemiologic survey over 22 consecutive years. J Perinat Med 32: 211–214. 15188792 10.1515/JPM.2004.039

[17] Rodier MH, Berthonneau J, Bourgoin A, Giraudeau G, Agius G, et al (1995) Seroprevalences of *Toxoplasma*, malaria, rubella, cytomegalovirus, HIV and treponemal infections among pregnant women in Cotonou, Republic of Benin. Acta Trop 59: 271–277. 8533662 10.1016/0001-706x(95)00087-u

[18] Fonseca AL, Silva RA, Fux B, Madureira AP, de Sousa FF, et al (2012) Epidemiologic aspects of toxoplasmosis and evaluation of its seroprevalence in pregnant women. Rev Soc Bras Med Trop 45: 357–364. 22760136 10.1590/s0037-86822012000300015

[19] Simpore J, Savadogo A, Ilboudo D, Nadambega MC, Esposito M, et al (2006) *Toxoplasma gondii*, HCV, and HBV seroprevalence and co-infection among HIV-positive and -negative pregnant women in Burkina Faso. J Med Virol 78: 730–733. 16628587 10.1002/jmv.20615

[20] Ndumbe PM, Andela A, Nkemnkengasong J, Watonsi E, Nyambi P (1992) Prevalence of infections affecting the child among pregnant women in Yaounde, Cameroon. Med Microbiol Immunol (Berl) 181: 127–130. 1522822 10.1007/BF00202052

[21] Many A, Koren G (2006) Toxoplasmosis during pregnancy. Can Fam Physician 52: : 29–30, 32. PMC1479740 16477906

[22] Rosso F, Les JT, Agudelo A, Villalobos C, Chaves JA, et al (2008) Prevalence of infection with *Toxoplasma gondii* among pregnant women in Cali, Colombia, South America. Am J Trop Med Hyg 78: 504–508. 18337350

[23] Arias ML, Chinchilla M, Reyes L, Linder E (1996) Seroepidemiology of toxoplasmosis in humans: possible transmission routes in Costa Rica. Rev Biol Trop 44: 377–381. 9246362

[24] Punda-Polic V, Tonkic M, Capkun V (2000) Prevalence of antibodies to *Toxoplasma gondii* in the female population of the County of Split Dalmatia, Croatia. Eur J Epidemiol 16: 875–877. 11297231 10.1023/a:1007606501923

[25] Sanchez-Gutierrez A, Martin-Hernandez I, Garcia-Izquierdo SM (2003) Estudio de reactividad a *Toxoplasma gondii* en embarazadas de las provincias Ciudad de la Habana y Pinar del Río, Cuba. Lab Enferm Infec 28: 3–8.

[26] Kaňková Š, Flegr J (2007) Longer pregnancy and slower fetal development in women with latent “asymptomatic” toxoplasmosis BMC Infect Dis 7: art: 114. 10.1186/1471-2334-7-114 PMC2233629 17916246

[27] Makuwa M, Lecko M, Nsimba B, Bakouetela J, Lounana-Kouta J (1992) Toxoplasmose et al femme enceinte au Congo Bilan de 5 ans de dépistage (1986–1990). Med Afr Noire 39: 493–495.

[28] Lebech M, Andersen O, Christensen NC, Hertel J, Nielsen HE, et al (1999) Feasibility of neonatal screening for *Toxoplasma* infection in the absence of prenatal treatment. Lancet 353: 1834–1837. 10359408 10.1016/s0140-6736(98)11281-3

[29] Attia RA, el-Zayat MM, Rizk H, Motawea S (1995) *Toxoplasma* IgG. & IgM. antibodies. A case control study. J Egypt Soc Parasitol 25: 877–882. 8586880

[30] Birgisdottir A, Asbjornsdottir H, Cook E, Gislason D, Jansson C, et al (2006) Seroprevalence of *Toxoplasma gondii* in Sweden, Estonia and Iceland. Scand J Infect Dis 38: 625–631. 16857606 10.1080/00365540600606556

[31] Dubey JP, Tiao N, Gebreyes WA, Jones JL (2012) A review of toxoplasmosis in humans and animals in Ethiopia. Epidemiol Infect 140: 1935–1938. 22874099 10.1017/S0950268812001392

[32] Koskiniemi M, Lappalainen M, Koskela P, Hedman K, Ammala P, et al.. (1992) The program for antenatal screening of toxoplasmosis in Finland: a prospective cohort study. Scand J Infect Dis Suppl 84: 70–74. 1290079

[33] Ancelle T, Goulet V, Tirard-Fleury V (2003) La toxoplasmose en France chez la femme enceinte en 2003: séroprévalence et facteurs associés. Bull Epidemiol Hebd 51: 227–229.

[34] Nabias R, Ngouamizokou A, Migot-Nabias F, Mbou-Moutsimbi RA, Lansoud-Soukate J (1998) [Serological investigation of toxoplasmosis in patients of the M.I.P. center of Franceville (Gabon)]. Bull Soc Pathol Exot Filial 91: 318–320. 9846226

[35] Fiedler K, Hulsse C, Straube W, Briese V (1999) [Toxoplasmosis-antibody seroprevalence in Mecklenburg-Western Pomerania]. Zentralbl Gynakol 121: 239–243. 10408076

[36] Antoniou M, Tzouvali H, Sifakis S, Galanakis E, Georgopoulou E, et al (2004) Incidence of toxoplasmosis in 5532 pregnant women in Crete, Greece: management of 185 cases at risk. Eur J Obstet Gynecol Reprod Biol 117: 138–143. 15541847 10.1016/j.ejogrb.2004.03.001

[37] Asthana SP, Macpherson CN, Weiss SH, Stephens R, Denny TN, et al (2006) Seroprevalence of *Toxoplasma gondii* in pregnant women and cats in Grenada, West Indies. J Parasitol 92: 644–645. 16884013 10.1645/GE-762R.1

[38] Szenasi Z, Horvath K, Sarkany E, Melles M (2005) Toxoplasmosis surveillance during pregnancy and quality assurance of methods in Hungary. Wien Klin Wochenschr 117: 29–34. 10.1007/s00508-005-0444-6 16416382

[39] Contreras MC, Schenone H, Salinas P, Sandoval L, Rojas A, et al (2009) Seroepidemiology of human toxoplasmosis in Chile. Rev Inst Med Trop Sao Paulo 38: 431–435. 10.1590/s0036-46651996000600008 9293090

[40] Liu Q, Wei F, Gao SY, Jiang L, Lian H, et al (2009) *Toxoplasma gondii* infection in pregnant women in China. T Roy Soc Trop Med H 103: 162–166. 10.1016/j.trstmh.2008.07.008 18822439

[41] Borkakoty BJ, Borthakur AK, Gohain M (2007) Prevalence of *Toxoplasma gondii* infection amongst pregnant women in Assam, India. Indian J Med Microbiol 25: 431–432. 18087109 10.4103/0255-0857.37365

[42] Konishi E, Houki Y, Harano K, Mibawani RS, Marsudi D, et al (2000) High prevalence of antibody to *Toxoplasma gondii* among humans in Surabaya, Indonesia. Japanese J Infect Dis 53: 238–241. 11227021

[43] Fallah M, Rabiee S, Matini M, Taherkhani H (2008) Seroepidemiology of toxoplasmosis in primigravida women in Hamadan, Islamic Republic of Iran, 2004. East Mediterr Health J 14: 163–171. 18557464

[44] Mahdi NK, Sharief M (2002) Risk factors for acquiring toxoplasmosis in pregnancy. J Bahrain Med Soc 14: 148–151.

[45] Ferguson W, Mayne PD, Lennon B, Butler K, Cafferkey M (2008) Susceptibility of pregnant women to *Toxoplasma* infection—potential benefits for newborn screening. Ir Med J 101: 220–221. 18807815

[46] Franklin DM, Dror Z, Nishri Z (1993) The prevalence and incidence of *Toxoplasma* antibodies in pregnant women. Isr J Med Sci 29: 285–286. 8314688

[47] De Paschale M, Agrappi C, Clerici P, Mirri P, Manco MT, et al (2008) Seroprevalence and incidence of *Toxoplasma gondii* infection in the Legnano area of Italy. Clin Microbiol Infect 14: 186–189. 18034857 10.1111/j.1469-0691.2007.01883.x

[48] Prabhakar P, Bailey A, Smikle MF, McCaw-Binns A, Ashley D (1991) Seroprevalence of *Toxoplasma gondii*, rubella virus, cytomegalovirus herpes simplex virus (TORCH) and syphilis in Jamaican pregnant women. West Indian Med J 40: 166–169. 1664562

[49] Sakikawa M, Noda S, Hanaoka M, Nakayama H, Hojo S, et al (2012) Anti-*Toxoplasma* antibody prevalence, primary infection rate, and risk factors in a study of toxoplasmosis in 4,466 pregnant women in Japan. Clin Vaccin Immunol 19: 365–367. 10.1128/CVI.05486-11 PMC3294603 22205659

[50] Jumaian NF (2005) Seroprevalence and risk factors for *Toxoplasma* infection in pregnant women in Jordan. East Mediterr Health J 11: 45–51. 16532670

[51] Iqbal J, Hira PR, Khalid N (2003) Toxoplasmosis in Kuwait: improved diagnosis based on quantitative immuno-assay. Clin Microbiol Infect 9: 336.

[52] Szenasi Z, Ozsvar Z, Nagy E, Jeszenszky M, Szabo J, et al (1997) Prevention of congenital toxoplasmosis in Szeged, Hungary. Int J Epidemiol 26: 428–435. 9169181 10.1093/ije/26.2.428

[53] Mousa DA, Mohammad MA, Toboli AB (2011) *Toxoplasma gondii* infection in pregnant women with previous adverse pregnancy outcome. Med J Islam World Acad Sci 19: 95–102.

[54] Rockiene L (1997) The prognosis of congenital toxoplasmosis in Lithuania. Hygiena Epidemiol 58: 39–45.

[55] Bobic B, Nikolic A, Klun I, Djurkovic-Djakovic O (2011) Kinetics of *Toxoplasma* infection in the Balkans. Wien Klin Wochenschr 123: 2–6. 21935646 10.1007/s00508-011-0052-6

[56] Lelong B, Rahelimino B, Candolfi E, Ravelojaona BJ, Villard O, et al (1995) [Prevalence of toxoplasmosis in a population of pregnant women in Antananarivo (Madagascar)]. Bull Soc Pathol Exot Filial 88: 46–49. 7787454

[57] Nissapatorn V, Noor Azmi MA, Cho SM, Fong MY, Init I, et al (2003) Toxoplasmosis: prevalence and risk factors. J Obstet Gynaecol 23: 618–624. 14617462 10.1080/01443610310001604376

[58] Caballero-Ortega H, Uribe-Salas FJ, Conde-Glez CJ, Cedillo-Pelaez C, Vargas-Villavicencio JA, et al (2012) Seroprevalence and national distribution of human toxoplasmosis in Mexico: analysis of the 2000 and 2006 National Health Surveys. T Roy Soc Trop Med H 106: 653–659. 10.1016/j.trstmh.2012.08.004 22998951

[59] El Mansouri B, Rhajaoui M, Sebti F, Amarir F, Laboudi M, et al (2007) [Seroprevalence of toxoplasmosis in pregnant women in Rabat, Morocco]. Bull Soc Pathol Exot Filial 100: 289–290. 17982862

[60] Sitoe SP, Rafael B, Meireles LR, Andrade HF Jr, Thompson R (2010) Preliminary report of HIV and *Toxoplasma gondii* occurrence in pregnant women from Mozambique. Rev Inst Med Trop Sao Paulo 52: 291–295. 21225211 10.1590/s0036-46652010000600002

[61] Rai SK, Shibata H, Sumi K, Rai G, Rai N, et al (1998) *Toxoplasma* antibody prevalence in Nepalese pregnant women and women with bad obstetric history. Southeast Asian J Trop Med Public Health 29: 739–743. 10772556

[62] Kortbeek LM, De Melker HE, Veldhuijzen IK, Conyn-Van Spaendonck MA (2004) Population-based *Toxoplasma* seroprevalence study in The Netherlands. Epidemiol Infect 132: 839–845. 15473146 10.1017/s0950268804002535 PMC2870170

[63] Morris A, Croxson M (2004) Serological evidence of *Toxoplasma gondii* infection among pregnant women in Auckland. N Z Med J 117: U770. 15014559

[64] Onadeko MO, Joynson DH, Payne RA (1992) The prevalence of *Toxoplasma* infection among pregnant women in Ibadan, Nigeria. J Trop Med Hyg 95: 143–145. 1560485

[65] Jenum PA, Kapperud G, Stray-Pedersen B, Melby KK, Eskild A, et al (1998) Prevalence of *Toxoplasma gondii* specific immunoglobulin G antibodies among pregnant women in Norway. Epidemiol Infect 120: 87–92. 9528822 10.1017/s0950268897008480 PMC2809341

[66] Ahmed MU, Hafiz A (1997) Toxoplasmosis and abortion: serological correlation. J Coll Phys Surg Pak 7: 156–159.

[67] Klufio CA, Delamare O, Amoa AB, Kariwiga G (1993) The prevalence of *Toxoplasma* antibodies in pregnant patients attending the Port Moresby General Hospital antenatal clinic: a seroepidemiological survey. P N G Med J 36: 4–9. 8266732

[68] Cantella R, Colichon A, Lopez L, Wu C, Goldfarb A, et al (1974) Toxoplasmosis in Peru. Geographic prevalence of *Toxoplasma gondii* antibodies in Peru studied by indirect fluorescent antibody technique. Trop Geogr Med 26: 204–209. 4605392

[69] Nowakowska D, Stray-Pedersen B, Spiewak E, Sobala W, Malafiej E, et al (2006) Prevalence and estimated incidence of *Toxoplasma* infection among pregnant women in Poland: a decreasing trend in the younger population. Clin Microbiol Infec 12: 913–917. 16882298 10.1111/j.1469-0691.2006.01513.x

[70] Lopes AP, Dubey JP, Moutinho O, Gargate MJ, Vilares A, et al (2012) Seroepidemiology of *Toxoplasma gondii* infection in women from the North of Portugal in their childbearing years. Epidemiol Infect 140: 872–877. 21878147 10.1017/S0950268811001658

[71] Abu-Madi MA, Behnke JM, Dabritz HA (2010) *Toxoplasma gondii* seropositivity and co-infection with TORCH pathogens in high-risk patients from Qatar. Am J Trop Med Hyg 82: 626–633. 20348511 10.4269/ajtmh.2010.09-0530 PMC2844547

[72] Lim H, Lee SE, Jung BK, Kim MK, Lee MY, et al (2012) Serologic survey of toxoplasmosis in Seoul and Jeju-do, and a brief review of its seroprevalence in Korea. Korean J Parasitol 50: 287–293. 23230325 10.3347/kjp.2012.50.4.287 PMC3514419

[73] Crucerescu E (1998) [Epidemiological data on toxoplasmosis. The aspects of congenital toxoplasmosis]. Bacteriol Virusol Parazitol Epidemiol 43: 147–155. 9932003

[74] Hung CC, Fan CK, Su KE, Sung FC, Chiou HY, et al (2007) Serological screening and toxoplasmosis exposure factors among pregnant women in the Democratic Republic of Sao Tome and Principe. Trans R Soc Trop Med Hyg 101: 134–139. 17113117 10.1016/j.trstmh.2006.04.012

[75] el Hady HM (1991) Toxoplasmosis among pregnant women in Abha, Saudi Arabia. J Egypt Soc Parasitol 21: 811–815. 1765694

[76] Faye O, Leye A, Dieng Y, Richard-Lenoble D, Diallo S (1998) [Toxoplasmosis in Dakar. Seroepidemiologic sampling of 353 women of reproductive age]. Bull Soc Pathol Exot Filial 91: 249–250. 9773203

[77] Wong A, Tan KH, Tee CS, Yeo GS (2000) Seroprevalence of cytomegalovirus, *Toxoplasma* and parvovirus in pregnancy. Singapore Med J 41: 151–155. 11063178

[78] Studenicova C, Ondriska F, Holkova R (2008) [Seroprevalence of *Toxoplasma gondii* among pregnant women in Slovakia]. Epidemiol Mikrobiol Imunol 57: 8–13. 18318393

[79] Logar J, Petrovec M, Novak-Antolic Z, Premru-Srsen T, Cizman M, et al (2002) Prevention of congenital toxoplasmosis in Slovenia by serological screening of pregnant women. Scand J Infect Dis 34: 201–204. 12030394 10.1080/00365540110080386

[80] Munoz Batet C, Guardia Llobet C, Juncosa Morros T, Vinas Domenech L, Sierra Soler M, et al (2004) [Toxoplasmosis and pregnancy. Multicenter study of 16,362 pregnant women in Barcelona]. Med Clin (Barc) 123: 12–16. 15207221 10.1016/s0025-7753(04)74396-1

[81] Elnahas A, Gerais AS, Elbashir MI, Eldien ES, Adam I (2003) Toxoplasmosis in pregnant Sudanese women. Saudi Med J 24: 868–870. 12939674

[82] Evengard B, Petersson K, Engman ML, Wiklund S, Ivarsson SA, et al (2001) Low incidence of *Toxoplasma* infection during pregnancy and in newborns in Sweden. Epidemiol Infect 127: 121–127. 11561964 10.1017/s0950268801005775 PMC2869718

[83] Signorell LM, Seitz D, Merkel S, Berger R, Rudin C (2006) Cord blood screening for congenital toxoplasmosis in northwestern Switzerland, 1982–1999. Pediatr Infect Dis J 25: 123–128. 16462288 10.1097/01.inf.0000195542.43369.96

[84] Doehring E, Reiter-Owona I, Bauer O, Kaisi M, Hlobil H, et al (1995) *Toxoplasma gondii* antibodies in pregnant women and their newborns in Dar es Salaam, Tanzania. Am J Trop Med Hyg 52: 546–548. 7611563 10.4269/ajtmh.1995.52.546

[85] Deniau M, Tourte-Schaefer C, Agbo K, Dupouy-Camet J, Heyer C, et al (1991) Évaluation des risques de toxoplasmose congénitale au Togo. Bull Soc Pathol Exot Filial 84: 664–672. 1819417

[86] Ramsewak S, Gooding R, Ganta K, Seepersadsingh N, Adesiyun AA (2008) Seroprevalence and risk factors of *Toxoplasma gondii* infection among pregnant women in Trinidad and Tobago. Rev Panam Salud Publica 23: 164–170. 18397582 10.1590/s1020-49892008000300003

[87] Ndong-Obame T, Ayadi A (1997) La toxoplasmose acquise et congénitale dans la région de Sfax (Tunisie). Bull Soc Fr Parasitol 15: 141–147.

[88] Harma M, Gungen N, Demir N (2004) Toxoplasmosis in pregnant women in Sanliurfa, Southeastern Anatolia City, Turkey. J Egypt Soc Parasitol 34: 519–525. 15287175

[89] Dar FK, Alkarmi T, Uduman S, Abdulrazzaq Y, Grundsell H, et al (1997) Gestational and neonatal toxoplasmosis: regional seroprevalence in the United Arab Emirates. Eur J Epidemiol 13: 567–571. 9258570 10.1023/a:1007392703037

[90] Nash JQ, Chissel S, Jones J, Warburton F, Verlander NQ (2005) Risk factors for toxoplasmosis in pregnant women in Kent, United Kingdom. Epidemiol Infect 133: 475–483. 15962554 10.1017/s0950268804003620 PMC2870271

[91] Jones JL, Kruszon-Moran D, Sanders-Lewis K, Wilson M (2007) *Toxoplasma gondii* infection in the United States, 1999-2004, decline from the prior decade. Am J Trop Med Hyg 77: 405–410. 17827351

[92] Triolo-Mieses M, Traviezo-Valles L (2006) Serological prevalence of *Toxoplasma gondii* antibodies in pregnancy in Palavecino Municipality Lara State,Venezuela. Kasmera 34: 7–13.

[93] Buchy P, Follezou JY, Lien TX, An TT, Tram LT, et al (2003) [Serological study of toxoplasmosis in Vietnam in a population of drug users (Ho Chi Minh city) and pregnant women (Nha Trang)]. Bull Soc Pathol Exot Filial 96: 46–47. 12784594

[94] Kodym P, Malý M, Švandová E, Lekatková H, Badoutová M, et al (2000) *Toxoplasma* in the Czech Republic 1923–1999: first case to widespread outbreak. Int J Parasitol 30: 11–18.

[95] Walton BC, De Arjona I, Benchoff BM (1966) Relationship of *Toxoplasma* antibodies to altitude. Am J Trop Med Hyg 15: 492–495. 5328733 10.4269/ajtmh.1966.15.492

[96] Jokelainen P, Nareaho A, Knaapi S, Oksanen A, Rikula U, et al (2010) *Toxoplasma gondii* in wild cervids and sheep in Finland: north-south gradient in seroprevalence. Vet Parasitol 171: 331–336. 20434266 10.1016/j.vetpar.2010.04.008

[97] Akanmu AS, Osunkalu VO, Ofomah JN, Olowoselu FO (2010) Pattern of demographic risk factors in the seroprevalence of anti-*Toxoplasma gondii* antibodies in HIV infected patients at the Lagos University Teaching Hospital. Nig Q J Hosp Med 20: 1–4. 20450022 10.4314/nqjhm.v20i1.57974

[98] Addebbous A, Adarmouch L, Tali A, Laboudi M, Amine M, et al (2012) IgG anti-*Toxoplasma* antibodies among asymptomatic HIV-infected patients in Marrakesh-Morocco. Acta Trop 123: 49–52. 22425678 10.1016/j.actatropica.2012.02.070

[99] Weight CM, Carding SR (2012) The protozoan pathogen *Toxoplasma gondii* targets the paracellular pathway to invade the intestinal epithelium. Ann N Y Acad Sci 1258: 135–142. 22731726 10.1111/j.1749-6632.2012.06534.x

[100] Lindsay DS, Dubey JP (2011) *Toxoplasma gondii*: the changing paradigm of congenital toxoplasmosis. Parasitology 138: 1829–1831. 21902872 10.1017/S0031182011001478

[101] Carneiro AC, Andrade GM, Costa JG, Pinheiro BV, Vasconcelos-Santos DV, et al (2013) Genetic characterization of *Toxoplasma gondii* revealed highly diverse genotypes for isolates from newborns with congenital toxoplasmosis in southeastern Brazil. J Clin Microbiol 51: 901–907. 23284022 10.1128/JCM.02502-12 PMC3592078

[102] Araujo FG, Slifer T (2003) Different strains of *Toxoplasma gondii* induce different cytokine responses in CBA/Ca mice. Infect Immun 71: 4171–4174. 12819111 10.1128/IAI.71.7.4171-4174.2003 PMC162004

[103] Nelson MM, Jones AR, Carmen JC, Sinai AP, Burchmore R, et al (2008) Modulation of the host cell proteome by the intracellular apicomplexan parasite *Toxoplasma gondii* . Infect Immun 76: 828–844. 17967855 10.1128/IAI.01115-07 PMC2223483

[104] Thirugnanam S, Rout N, Gnanasekar M (2013) Possible role of *Toxoplasma gondii* in brain cancer through modulation of host microRNAs. Infect Agent Cancer 8: 8. 23391314 10.1186/1750-9378-8-8 PMC3583726

[105] Dubey JP, Ferreira LR, Martins J, McLeod R (2012) Oral oocyst-induced mouse model of toxoplasmosis: effect of infection with *Toxoplasma gondii* strains of different genotypes, dose, and mouse strains (transgenic, out-bred, in-bred) on pathogenesis and mortality. Parasitology 139: 1–13. 22078010 10.1017/S0031182011001673 PMC3683600

[106] McLeod R, Boyer KM, Lee D, Mui E, Wroblewski K, et al (2012) Prematurity and severity are associated with *Toxoplasma gondii* alleles (NCCCTS, 1981–2009). Clin Infect Dis 54: 1595–1605. 22499837 10.1093/cid/cis258 PMC3348955

[107] Khan A, Fux B, Su C, Dubey JP, Darde ML, et al (2007) Recent transcontinental sweep of *Toxoplasma gondii* driven by a single monomorphic chromosome. Proc Natl Acad Sci USA 104: 14872–14877. 17804804 10.1073/pnas.0702356104 PMC1965483

[108] Mercier A, Devillard S, Ngoubangoye B, Bonnabau H, Banuls AL, et al (2010) Additional haplogroups of *Toxoplasma gondii* out of Africa: population structure and mouse-virulence of strains from Gabon. PLoS Neglect Trop D 4: e876. 10.1371/journal.pntd.0000876 PMC2970538 21072237

[109] Khan A, Jordan C, Muccioli C, Vallochi AL, Rizzo LV, et al (2006) Genetic divergence of *Toxoplasma gondii* strains associated with ocular toxoplasmosis, Brazil. Emerg Infect Dis 12: 942–949. 16707050 10.3201/eid1206.060025 PMC3373049

[110] Glasner PD, Silveira C, Kruszon-Moran D, Martins MC, Burnier Junior M, et al (1992) An unusually high prevalence of ocular toxoplasmosis in southern Brazil. Am J Ophthalmol 114: 136–144. 1642287 10.1016/s0002-9394(14)73976-5

[111] Frenkel JK (1953) Host, strain and treatment variation as factors in the pathogenesis of toxoplasmosis. Am J Trop Med Hyg 2: 390–415. 13040675 10.4269/ajtmh.1953.2.390

[112] Jacobs L (1956) Propagation, morphology, and biology of *Toxoplasma* . Ann N Y Acad Sci 64: 154–179.

[113] Prestrud KW, Asbakk K, Oksanen A, Nareaho A, Jokelainen P (2010) *Toxoplasma gondii* in the Subarctic and Arctic. Acta Vet Scand (Suppl. 1): 57.

[114] Sroka J (2001) Seroepidemiology of toxoplasmosis in the Lublin region. Ann Agric Environ Med 8: 25–31. 11426921

[115] Petersen E, Vesco G, Villari S, Buffolano W (2010) What do we know about risk factors for infection in humans with *Toxoplasma gondii* and how can we prevent infections? Zoonoses Public Health 57: 8–17. 19744301 10.1111/j.1863-2378.2009.01278.x

[116] Alvarado-Esquivel C, Alanis-Quinones OP, Arreola-Valenzuela MA, Rodriguez-Briones A, Piedra-Nevarez LJ, et al (2006) Seroepidemiology of *Toxoplasma gondii* infection in psychiatric inpatients in a northern Mexican city. BMC Infect Dis 6: 178. 17178002 10.1186/1471-2334-6-178 PMC1764421

[117] Ferguson DJ (2009) Identification of faecal transmission of *Toxoplasma gondii*: Small science, large characters. Int J Parasitol 39: 871–875. 19596630 10.1016/j.ijpara.2009.01.003

[118] Kolbeková P, Kourbatova E, Novotná M, Kodym P, Flegr J (2007) New and old risk-factors for *Toxoplasma gondii* infection: prospective cross-sectional study among military personnel in the Czech Republic. Clin Microbiol Infec 13: 1012–1017. 17617185 10.1111/j.1469-0691.2007.01771.x

[119] Jones JL, Dargelas V, Roberts J, Press C, Remington JS, et al (2009) Risk factors for *Toxoplasma gondii* infection in the United States. Clin Infect Dis 49: 878–884. 19663709 10.1086/605433

[120] Yazar S, Eser B, Yay M (2006) Prevalence of anti-*Toxoplasma gondii* antibodies in Turkish blood donors. Ethiop Med J 44: 257–261. 17447392

[121] Elhence P, Agarwal P, Prasad KN, Chaudhary RK (2010) Seroprevalence of *Toxoplasma gondii* antibodies in North Indian blood donors: Implications for transfusion transmissible toxoplasmosis. Transfus Apher Sci 43: 37–40. 20605111 10.1016/j.transci.2010.05.004

[122] Silveira C, Vallochi AL, da Silva UR, Muccioli C, Holland GN, et al (2011) *Toxoplasma gondii* in the peripheral blood of patients with acute and chronic toxoplasmosis. Br J Ophthalmol 95: 396–400. 20601663 10.1136/bjo.2008.148205

[123] Hafid J, Bellete B, Flori P, Sawadogo P, Boyer Y (2005) Materno-foetal transmission of murine toxoplasmosis after oral infection. Am J Immunol 1: 1–5.

[124] Schroder J, Tiilikainen A, De la Chapelle A (1974) Fetal leukocytes in the maternal circulation after delivery. I. Cytological aspects. Transplantation 17: 346–354. 4823382

[125] Fischer SA (2006) Infections complicating solid organ transplantation. Surg Clin North Am 86: : 1127–1145, v-vi. 10.1016/j.suc.2006.06.005 16962405

[126] Derouin F, Pelloux H (2008) Prevention of toxoplasmosis in transplant patients. Clin Microbiol Infect 14: 1089–1101. 19018809 10.1111/j.1469-0691.2008.02091.x

[127] Edvinsson B, Lundquist J, Ljungman P, Ringden O, Evengard B (2008) A prospective study of diagnosis of *Toxoplasma gondii* infection after bone marrow transplantation. APMIS 116: 345–351. 18452424 10.1111/j.1600-0463.2008.00871.x

[128] Fricker-Hidalgo H, Bulabois CE, Brenier-Pinchart MP, Hamidfar R, Garban F, et al (2009) Diagnosis of toxoplasmosis after allogeneic stem cell transplantation: results of DNA detection and serological techniques. Clin Infect Dis 48: e9–e15. 19072243 10.1086/595709

[129] Laibe S, Ranque S, Curtillet C, Faraut F, Dumon H, et al (2006) Timely diagnosis of disseminated toxoplasmosis by sputum examination. J Clin Microbiol 44: 646–648. 16455936 10.1128/JCM.44.2.646-648.2006 PMC1392660

[130] Hiramoto RM, Mayrbaurl-Borges M, Galisteo AJ Jr, Meireles LR, Macre MS, et al (2001) Infectivity of cysts of the ME-49 *Toxoplasma gondii* strain in bovine milk and homemade cheese. Rev Saude Publica 35: 113–118. 11359195 10.1590/s0034-89102001000200002

[131] Camossi LG, Greca-Junior H, Correa AP, Richini-Pereira VB, Silva RC, et al (2011) Detection of *Toxoplasma gondii* DNA in the milk of naturally infected ewes. Vet Parasitol 177: 256–261. 21216534 10.1016/j.vetpar.2010.12.007

[132] Arantes TP, Lopes WDZ, Ferreira RM, Pieroni JSP, Pinto VMR, et al (2009) *Toxoplasma gondii*: Evidence for the transmission by semen in dogs. Exp Parasitol 123: 190–194. 19622353 10.1016/j.exppara.2009.07.003

[133] Singh S, Singh N (1993) Toxoplasmosis is transmitted sexually. Int Conf AIDS 9: 490.

[134] Elsheikha HM (2008) Congenital toxoplasmosis: Priorities for further health promotion action. Public Health 122: 335–353. 17964621 10.1016/j.puhe.2007.08.009

[135] Joiner KA, Dubremetz JF (1993) *Toxoplasma gondii*: a protozoan for the nineties. Infect Immun 61: 1169–1172. 8454321 10.1128/iai.61.4.1169-1172.1993 PMC281344

[136] Channon JY, Seguin RM, Kasper LH (2000) Differential infectivity and division of *Toxoplasma gondii* in human peripheral blood leukocytes. Infect Immun 68: 4822–4826. 10899898 10.1128/iai.68.8.4822-4826.2000 PMC98447

[137] Lambert H, Hitziger N, Dellacasa I, Svensson M, Barragan A (2006) Induction of dendritic cell migration upon *Toxoplasma gondii* infection potentiates parasite dissemination. Cell Microbiol 8: 1611–1623. 16984416 10.1111/j.1462-5822.2006.00735.x

[138] Da Gama LM, Ribeiro-Gomes FL, Guimaraes U Jr, Arnholdt AC (2004) Reduction in adhesiveness to extracellular matrix components, modulation of adhesion molecules and in vivo migration of murine macrophages infected with *Toxoplasma gondii* . Microbes Infect 6: 1287–1296. 15555535 10.1016/j.micinf.2004.07.008

[139] Courret N, Darche S, Sonigo P, Milon G, Buzoni-Gatel D, et al (2006) CD11c- and CD11b-expressing mouse leukocytes transport single *Toxoplasma gondii* tachyzoites to the brain. Blood 107: 309–316. 16051744 10.1182/blood-2005-02-0666 PMC1895351

[140] Lambert H, Vutova PP, Adams WC, Lore K, Barragan A (2009) The *Toxoplasma gondii*-shuttling function of dendritic cells is linked to the parasite genotype. Infect Immun 77: 1679–1688. 19204091 10.1128/IAI.01289-08 PMC2663171

[141] Bierly AL, Shufesky WJ, Sukhumavasi W, Morelli AE, Denkers EY (2008) Dendritic cells expressing plasmacytoid marker PDCA-1 are Trojan horses during *Toxoplasma gondii* infection. J Immunol 181: 8485–8491. 19050266 10.4049/jimmunol.181.12.8485 PMC2626190

[142] Lambert H, Barragan A (2010) Modelling parasite dissemination: host cell subversion and immune evasion by *Toxoplasma gondii* . Cell Microbiol 12: 292–300. 19995386 10.1111/j.1462-5822.2009.01417.x

[143] Persson CM, Lambert H, Vutova PP, Dellacasa-Lindberg I, Nederby J, et al (2009) Transmission of *Toxoplasma gondii* from infected dendritic cells to natural killer cells. Infect Immun 77: 970–976. 19139191 10.1128/IAI.00833-08 PMC2643636

[144] Nigro G, Piazze J, Paesano R, Mango T, Provvedi S, et al (1999) Low levels of natural killer cells in pregnant women transmitting *Toxoplasma gondii* . Prenat Diagn 19: 401–404. 10360506

[145] Montoya JG, Liesenfeld O (2004) Toxoplasmosis. Lancet 363: 1965–1975. 15194258 10.1016/S0140-6736(04)16412-X

[146] Vaillant V, de Valk H, Baron E, Ancelle T, Colin P, et al (2005) Foodborne infections in France. Foodborne Pathog Dis 2: 221–232. 16156703 10.1089/fpd.2005.2.221

[147] Mead PS, Slutsker L, Dietz V, McCaig LF, Bresee JS, et al (1999) Food-related illness and death in the United States. Emerg Infect Dis 5: 607–625. 10511517 10.3201/eid0505.990502 PMC2627714

[148] Klaren VNA, Kijlstra A (2002) Toxoplasmosis, an overview with emphasis on ocular involvement. Ocul Immunol Inflamm 10: 1–26. 12461700 10.1076/ocii.10.1.1.10330

[149] Havelaar AH, Kemmeren JM, Kortbeek LM (2007) Disease burden of congenital toxoplasmosis. Clin Infect Dis 44: 1467–1474. 17479945 10.1086/517511

[150] Gilbert RE, Stanford MR (2000) Is ocular toxoplasmosis caused by prenatal or postnatal infection? Br J Ophthalmol 84: 224–226. 10655202 10.1136/bjo.84.2.224 PMC1723371

[151] Jones JL, Holland GN (2010) Annual burden of ocular toxoplasmosis in the US. Am J Trop Med Hyg 82: 464–465. 20207874 10.4269/ajtmh.2010.09-0664 PMC2829910

[152] Porter SB, Sande MA (1992) Toxoplasmosis of the central nervous system in the acquired immunodeficiency syndrome. N Engl J Med 327: 1643–1648. 1359410 10.1056/NEJM199212033272306

[153] Beaver PC, Jung RC, Cupp EW, Craig CF (1984) Clinical Parasitology. Philadelphia: Lea & Febiger. viii, 825 p. p.

[154] Carter CJ (2013) Toxoplasmosis and polygenic disease susceptibility genes: extensive *Toxoplasma gondii* host/pathogen interactome enrichment in nine psychiatric or neurological disorders. J Pathog 2013: 965046. 23533776 10.1155/2013/965046 PMC3603208

[155] Ajzenberg D, Cogne N, Paris L, Bessieres MH, Thulliez P, et al (2002) Genotype of 86 *Toxoplasma gondii* isolates associated with human congenital toxoplasmosis, and correlation with clinical findings. J Infect Dis 186: 684–689. 12195356 10.1086/342663

[156] Weiss LM, Dubey JP (2009) Toxoplasmosis: A history of clinical observations. Int J Parasitol 39: 895–901. 19217908 10.1016/j.ijpara.2009.02.004 PMC2704023

[157] Robert-Gangneux F, Gangneux JP, Vu N, Jaillard S, Guiguen C, et al (2011) High level of soluble HLA-G in amniotic fluid is correlated with congenital transmission of *Toxoplasma gondii* . Clin Immunol 138: 129–134. 21185786 10.1016/j.clim.2010.12.004

[158] Silveira C, Ferreira R, Muccioli C, Nussenblatt R, Belfort R (2003) Toxoplamosis transmitted to a newborn from the mother infected 20 years earlier. Am J Ophthalmol 136: 370–371. 12888070 10.1016/s0002-9394(03)00191-0

[159] Hinze-Selch D, Daubener W, Erdag S, Wilms S (2010) The diagnosis of a personality disorder increases the likelihood for seropositivity to *Toxoplasma gondii* in psychiatric patients. Folia Parasitol 57: 129–135. 10.14411/fp.2010.016 20608475

[160] Zhu S (2009) Psychosis may be associated with toxoplasmosis. Med Hypotheses 73: 799–801. 19467790 10.1016/j.mehy.2009.04.013

[161] Okusaga O, Langenberg P, Sleemi A, Vaswani D, Giegling I, et al (2011) *Toxoplasma gondii* antibody titers and history of suicide attempts in patients with schizophrenia. Schizophr Res 133: 150–155. 21890329 10.1016/j.schres.2011.08.006

[162] Emelia O, Amal RN, Ruzanna ZZ, Shahida H, Azzubair Z, et al (2012) Seroprevalence of anti-*Toxoplasma gondii* IgG antibody in patients with schizophrenia. Trop Biomed 29: 151–159. 22543615

[163] Mortensen PB, Pedersen CB, McGrath JJ, Hougaard DM, Norgaard-Petersen B, et al (2011) Neonatal antibodies to infectious agents and risk of bipolar disorder: a population-based case-control study. Bipolar Disorder 13: 624–629. 10.1111/j.1399-5618.2011.00962.x 22085475

[164] Blomstrom A, Karlsson H, Wicks S, Yang S, Yolken RH, et al (2012) Maternal antibodies to infectious agents and risk for non-affective psychoses in the offspring—a matched case-control study. Schizophr Res 140: 25–30. 22819777 10.1016/j.schres.2012.06.035

[165] Tedla Y, Shibre T, Ali O, Tadele G, Woldeamanuel Y, et al (2011) Serum antibodies to *Toxoplasma gondii* and Herpesviridae family viruses in individuals with schizophrenia and bipolar disorder: a case-control study. Ethiop Med J 49: 211–220. 21991754

[166] Torrey EF, Bartko JJ, Lun ZR, Yolken RH (2007) Antibodies to *Toxoplasma gondii* in patients with schizophrenia: A meta-analysis. Schizophr Bull 33: 729–736. 17085743 10.1093/schbul/sbl050 PMC2526143

[167] Pearce BD, Kruszon-Moran D, Jones JL (2012) The relationship between *Toxoplasma gondii* infection and mood disorders in the Third National Health and Nutrition Survey. Biol Psychiatry 72: 290–295. 22325983 10.1016/j.biopsych.2012.01.003 PMC4750371

[168] Groer MW, Yolken RH, Xiao JC, Beckstead JW, Fuchs D, et al. (2011) Prenatal depression and anxiety in *Toxoplasma gondii*-positive women. Am J Obstet Gynecol 204.. 10.1016/j.ajog.2011.01.004 PMC3144318 21345406

[169] Radford A, Williams SN, Kane B, Groer M (2012) Relationships of *Toxoplasma* antibody titers and dysphoric moods in female veterans. Brain, Behaviour, and Immunity 26..

[170] Yagmur F, Yazar S, Temel HO, Cavusoglu M (2010) May *Toxoplasma gondii* increase suicide attempt - preliminary results in Turkish subjects? Forensic Sci Int 199: 15–17. 20219300 10.1016/j.forsciint.2010.02.020

[171] Pedersen MG, Mortensen PB, Norgaard-Pedersen B, Postolache TT (2012) *Toxoplasma gondii* infection and self-directed violence in mothers. Arch Gen Psychiatry 69: 1123–1130. 22752117 10.1001/archgenpsychiatry.2012.668

[172] Lester D (2010) Predicting European suicide rates with physiological indices. Psychol Rep 107: 713–714. 21323128 10.2466/12.19.PR0.107.6.713-714

[173] Ling VJ, Lester D, Mortensen PB, Postolache TT (2011) *Toxoplasma gondii* seropositivity and completed suicide in 20 European countries. Biol Psychiatry 69: 500.

[174] Lester D (2012) *Toxoplasma gondii* and homicide. Psychol Rep 111: 196–197. 23045862 10.2466/12.15.16.PR0.111.4.196-197

[175] Brynska A, Tomaszewicz-Libudzic E, Wolanczyk T (2001) Obsessive-compulsive disorder and acquired toxoplasmosis in two children. Eur Child Adolesc Psychiatry 10: 200–204. 11596821 10.1007/s007870170027

[176] Miman O, Mutlu EA, Ozcan O, Atambay M, Karlidag R, et al (2010) Is there any role of *Toxoplasma gondii* in the etiology of obsessive-compulsive disorder? Psychiatry Res 177: 263–265. 20106536 10.1016/j.psychres.2009.12.013

[177] Parness-Yossifon R, Margalit D, Pollack A, Leiba H (2008) Behavioral disorders in children with idiopathic intracranial hypertension. J Child Neurol 23: 447–450. 18401035 10.1177/0883073807308709

[178] Stahl W, Turek G (1988) Chronic murine toxoplasmosis: clinicopathologic characterization of a progressive wasting syndrome. Ann Trop Med Parasitol 82: 35–48. 3401069 10.1080/00034983.1988.11812206

[179] Arsenijevic D, Girardier L, Seydoux J, Pechere JC, Garcia I, et al (1998) Metabolic-cytokine responses to a second immunological challenge with LPS in mice with *T. gondii* infection. Am J Physiol 274: E439–445. 9530126 10.1152/ajpendo.1998.274.3.E439

[180] Arsenijevic D, Bilbao FD, Giannakopoulos P, Girardier L, Samec S, et al (2001) A role for interferon-gamma in the hypermetabolic response to murine toxoplasmosis. Eur Cytokine Netw 12: 518–527. 11566633

[181] Arsenijevic D, Girardier L, Seydoux J, Chang HR, Dulloo AG (1997) Altered energy balance and cytokine gene expression in a murine model of chronic infection with *Toxoplasma gondii* American Journal of Physiology-Endocrinology and Metabolism. 35: 908–917. 10.1152/ajpendo.1997.272.5.E908 9176193

[182] Prandota J (2011) Metabolic, immune, epigenetic, endocrine and phenotypic abnormalities found in individuals with autism spectrum disorders, Down syndrome and Alzheimer disease may be caused by congenital and/or acquired chronic cerebral toxoplasmosis. Res Autism Spectr Disorders 5: 14–59.

[183] Prandota J (2010) Autism spectrum disorders may be due to cerebral toxoplasmosis associated with chronic neuroinflammation causing persistent hypercytokinemia that resulted in an increased lipid peroxidation, oxidative stress, and depressed metabolism of endogenous and exogenous substances. Res Autism Spectr Disorders 4: 119–155.

[184] Prandota J (2009) Neuropathological changes and clinical features of autism spectrum disorder participants are similar to that reported in congenital and chronic cerebral toxoplasmosis in humans and mice. Res Autism Spectr Disorders 4: 103–118.

[185] Conley FK, Jenkins KA (1981) Immunohistological study of the anatomic relationship of *Toxoplasma* antigens to the inflammatory response in the brains of mice chronically infected with *Toxoplasma gondii* Infect Immun. 31: 1184–1192. 10.1128/iai.31.3.1184-1192.1981 PMC351441 7228401

[186] Ribeiro DA, Pereira PCM, Machado JM, Silva SB, Pessoa AWP, et al (2004) Does toxoplasmosis cause DNA damage? An evaluation in isogenic mice under normal diet or dietary restriction. Mutat Res- Gen Tox En 559: 169–176. 10.1016/j.mrgentox.2004.01.007 15066584

[187] Massimine KM, Doan LT, Atreya CA, Stedman TT, Anderson KS, et al (2005) *Toxoplasma gondii* is capable of exogenous folate transport - A likely expansion of the BT1 family of transmembrane proteins. Mol Biochem Parasitol 144: 44–54. 16159678 10.1016/j.molbiopara.2005.07.006

[188] Al-Gazali LI, Padmanabhan R, Melnyk S, Yi P, Pogribny IP, et al (2001) Abnormal folate metabolism and genetic polymorphism of the folate pathway in a child with Down syndrome and neural tube defect. Am J Med Genet 103: 128–132. 11568918 10.1002/ajmg.1509

[189] Rao AA, Sridhar GR, Das UN (2007) Elevated butyrylcholinesterase and acetylcholinesterase may predict the development of type 2 diabetes mellitus and Alzheimer's disease. Med Hypotheses 69: 1272–1276. 17553629 10.1016/j.mehy.2007.03.032

[190] Kusbeci OY, Miman O, Yaman M, Aktepe OC, Yazar S (2011) Could *Toxoplasma gondii* have any role in Alzheimer disease? Alzheimer Dis Assoc Disord 25: 1–3. 20921875 10.1097/WAD.0b013e3181f73bc2

[191] Chan WF, Gurnot C, Montine TJ, Sonnen JA, Guthrie KA, et al (2012) Male microchimerism in the human female brain. PLoS ONE 7: e45592. 23049819 10.1371/journal.pone.0045592 PMC3458919

[192] Miman O, Kusbeci OY, Aktepe OC, Cetinkaya Z (2010) The probable relation between *Toxoplasma gondii* and Parkinson's disease. Neurosci Lett 475: 129–131. 20350582 10.1016/j.neulet.2010.03.057

[193] Murakami T, Nakajima M, Nakamura T, Hara A, Uyama E, et al (2000) Parkinsonian symptoms as an initial manifestation in a Japanese patient with acquired immunodeficiency syndrome and *Toxoplasma* infection. Intern Med 39: 1111–1114. 11197803 10.2169/internalmedicine.39.1111

[194] Koseoglu E, Yazar S, Koc I (2009) Is *Toxoplasma gondii* a causal agent in migraine? Am J Med Sci 338: 120–122. 19564786 10.1097/MAJ.0b013e31819f8cac

[195] Prandota J (2010) Migraine associated with patent foramen ovale may be caused by reactivation of cerebral toxoplasmosis triggered by arterial blood oxygen desaturation. Int J Neurosci 120: 81–87. 20199198 10.3109/00207450903458647

[196] Prandota J (2009) The importance of *Toxoplasma gondii* infection in diseases presenting with headaches. Headaches and aseptic meningitis may be manifestations of the Jarisch-Herxheimer reaction. Int J Neurosci 119: 2144–2182. 19916846 10.3109/00207450903149217

[197] Prandota J (2007) Recurrent headache as the main symptom of acquired cerebral toxoplasmosis in nonhuman immunodeficiency virus-infected subjects with no lymphadenopathy: the parasite may be responsible for the neurogenic inflammation postulated as a cause of different types of headaches. Am J Ther 14: 63–105. 17303977 10.1097/01.mjt.0000208272.42379.aa

[198] Kusbeci OY, Miman O, Yaman M, Aktepe OC, Yazar S (2011) Could *Toxoplasma gondii* have any role in Alzheimer disease? Alzheimer Dis Assoc Disord 25: 1–3. 20921875 10.1097/WAD.0b013e3181f73bc2

[199] Prandota J (2009) Mollaret meningitis may be caused by reactivation of latent cerebral toxoplasmosis. Int J Neurosci 119: 1655–1692. 19922380 10.1080/00207450802480044

[200] Palmer BS (2007) Meta-analysis of three case controlled studies and an ecological study into the link between cryptogenic epilepsy and chronic toxoplasmosis infection. Seizure 16: 657–663. 17604653 10.1016/j.seizure.2007.05.010

[201] Stommel EW, Seguin R, Thadani VM, Schwartzman JD, Gilbert K, et al (2001) Cryptogenic epilepsy: an infectious etiology? Epilepsia 42: 436–438. 11442166 10.1046/j.1528-1157.2001.25500.x

[202] Michaoowicz R, Jozwiak S, Ignatowicz R, Szwabowska-Orzeszko E (1988) Landau-Kleffner syndrome—epileptic aphasia in children—possible role of *Toxoplasma gondii* infection. Acta Paediatr Hung 29: 337–342. 2479399

[203] Riga M, Kefalidis G, Chatzimoschou A, Tripsianis G, Kartali S, et al (2011) Increased seroprevalence of *Toxoplasma gondii* in a population of patients with Bell's palsy: a sceptical interpretation of the results regarding the pathogenesis of facial nerve palsy. Eur Arch Otorhinolaryngol 268: 1087–1092. 21305313 10.1007/s00405-011-1499-9

[204] Andrade GM, Resende LM, Goulart EM, Siqueira AL, Vitor RW, et al (2008) Hearing loss in congenital toxoplasmosis detected by newborn screening. Braz J Otorhinolaryngol 74: 21–28. 18392497 10.1016/S1808-8694(15)30746-1 PMC9450616

[205] al Muhaimeed H (1996) Prevalence of sensorineural hearing loss due to toxoplasmosis in Saudi children: a hospital based study. Int J Pediatr Otorhinolaryngol 34: 1–8. 8770668 10.1016/0165-5876(95)01223-0

[206] Yamakawa R, Yamashita Y, Yano A, Morita J, Kato H (1996) Congenital toxoplasmosis complicated by central diabetes insipidus in an infant with Down syndrome. Brain Dev 18: 75–77. 8907349 10.1016/0387-7604(95)00099-2

[207] Oygur N, Yilmaz G, Ozkaynak C, Guven AG (1998) Central diabetes insipitus in a patient with congenital toxoplasmosis. Am J Perinatol 15: 191–192. 9572376 10.1055/s-2007-993924

[208] Nitta A, Suzumura H, Kano K, Arisaka O (2006) Congenital toxoplasmosis complicated with central diabetes insipidus in the first week of life. J Pediatr 148: 283. 10.1016/j.jpeds.2005.05.030 16492446

[209] Gherardi R, Baudrimont M, Lionnet F, Salord JM, Duvivier C, et al (1992) Skeletal muscle toxoplasmosis in patients with acquired immunodeficiency syndrome: a clinical and pathological study. Ann Neurol 32: 535–542. 1456737 10.1002/ana.410320409

[210] Massa G, Vanderschueren-Lodeweyckx M, Van Vliet G, Craen M, de Zegher F, et al (1989) Hypothalamo-pituitary dysfunction in congenital toxoplasmosis. Eur J Pediatr 148: 742–744. 2792124 10.1007/BF00443099

[211] Siahanidou T, Tsoumas D, Kanaka-Gantenbein C, Mandyla H (2006) Neuroendocrine abnormalities in a neonate with congenital toxoplasmosis. J Pediatr Endocrinol Metab 19: 1363–1366. 17220066 10.1515/jpem.2006.19.11.1363

[212] Wrensch M, Bondy ML, Wiencke J, Yost M (1993) Environmental risk factors for primary malignant brain tumors: a review. J Neurooncol 17: 47–64. 8120572 10.1007/BF01054274

[213] Ryan P, Hurley SF, Johnson AM, Salzberg M, Lee MW, et al (1993) Tumours of the brain and presence of antibodies to *Toxoplasma gondii* . Int J Immunol 22: 412–419. 10.1093/ije/22.3.412 8359956

[214] Thomas F, Lafferty KD, Brodeur J, Elguero E, Gauthier-Clerc M, et al (2012) Incidence of adult brain cancers is higher in countries where the protozoan parasite *Toxoplasma gondii* is common. Biol Lett 8: 101–103. 21795265 10.1098/rsbl.2011.0588 PMC3259962

[215] Vittecoq M, Elguero E, Lafferty KD, Roche B, Brodeur J, et al (2012) Brain cancer mortality rates increase with *Toxoplasma gondii* seroprevalence in France. Infect Genet Evol 12: 496–498. 22285308 10.1016/j.meegid.2012.01.013

[216] Johnson MD, Jennings MT, Gold LI, Moses HL (1993) Transforming growth factor-beta in neural embryogenesis and neoplasia. Hum Pathol 24: 457–462. 8387957 10.1016/0046-8177(93)90156-b

[217] Ortiz-Munoz AB, Cuadrado-Mendez L, Sanchis-Belenguer R (1984) [Possible interactions between *Toxoplasma gondii* infection and the presence of non-Hodgkin's lymphoma]. Rev Esp Oncol 31: 237–245. 6400214

[218] Herold MA, Kuhne R, Vosberg M, Ostheeren-Michaelis S, Vogt P, et al (2009) Disseminated toxoplasmosis in a patient with non-Hodgkin lymphoma. Infection 37: 551–554. 19499181 10.1007/s15010-009-9007-5

[219] Yazar S, Yaman O, Eser B, Altuntas F, Kurnaz F, et al (2004) Investigation of anti-*Toxoplasma gondii* antibodies in patients with neoplasia. J Med Microbiol 53: 1183–1186. 15585495 10.1099/jmm.0.45587-0

[220] Alibek K, Kakpenova A, Baiken Y (2013) Role of infectious agents in the carcinogenesis of brain and head and neck cancers. Infect Agent Cancer 8: 7. 23374258 10.1186/1750-9378-8-7 PMC3573938

[221] Grudzien M (1968) [*Toxoplasma* reactions in women with neoplasms]. Pol Tyg Lek 23: 54–56. 5654432

[222] Gupta A, Driscoll MS (2010) Do hormones influence melanoma? Facts and controversies. Clin Dermatol 28: 287–292. 20541681 10.1016/j.clindermatol.2010.04.003

[223] Nguyen Huu S, Oster M, Avril MF, Boitier F, Mortier L, et al (2009) Fetal microchimeric cells participate in tumour angiogenesis in melanomas occurring during pregnancy. Am J Pathol 174: 630–637. 19147820 10.2353/ajpath.2009.080566 PMC2630570

[224] Kim DS, Park SH, Park KC (2004) Transforming growth factor-beta1 decreases melanin synthesis via delayed extracellular signal-regulated kinase activation. Int J Biochem Cell Biol 36: 1482–1491. 15147727 10.1016/j.biocel.2003.10.023

[225] Nagineni CN, Detrick B, Hooks JJ (2002) Transforming growth factor-beta expression in human retinal pigment epithelial cells is enhanced by *Toxoplasma gondii*: a possible role in the immunopathogenesis of retinochoroiditis. Clin Exp Immunol 128: 372–378. 11985530 10.1046/j.1365-2249.2002.01815.x PMC1906397

[226] Connor TB Jr, Roberts AB, Sporn MB, Danielpour D, Dart LL, et al (1989) Correlation of fibrosis and transforming growth factor-beta type 2 levels in the eye. J Clin Invest 83: 1661–1666. 2708527 10.1172/JCI114065 PMC303874

[227] Vos GH (1987) Population studies showing cross-reactivity of *Toxoplasma gondii* antibodies with antibodies to malignant cervical tissue antigens. S Afr Med J 71: 78–82. 3810351

[228] Sanchis-Belenguer R, Cuadrado-Mendez L, Ortiz Munoz AB (1984) [Possible interactions between *Toxoplasma gondii* infection and the presence of carcinomas of female genitalia and the breast]. Rev Esp Oncol 31: 247–255. 6545708

[229] Yazar S, Gur M, Ozdogru I, Yaman O, Oguzhan A, et al (2006) Anti-*Toxoplasma gondii* antibodies in patients with chronic heart failure. J Med Microbiol 55: 89–92. 16388035 10.1099/jmm.0.46255-0

[230] Lappin MR (2010) Update on the diagnosis and management of *Toxoplasma gondii* infection in cats. Top Companion Anim Med 25: 136–141. 20937495 10.1053/j.tcam.2010.07.002

[231] Paspalaki PK, Mihailidou EP, Bitsori M, Tsagkaraki D, Mantzouranis E (2001) Polyomyositis and myocarditis associated with acquired toxoplasmosis in an immunocompetent girl. BMC Musculoskelet Disord 2: 8. 11818029 10.1186/1471-2474-2-8 PMC65052

[232] Prandota J (2012) Gastrointestinal tract abnormalities in autism, inflammatory bowel disease and many other clinical entities may be due to *T. gondii* infection. Open Acc Sci Rep 1: 256.

[233] Arciszewski M, Pierzynowski S, Ekblad E (2005) Lipopolysaccharide induces cell death in cultured porcine myenteric neurons. Dig Dis Sci 50: 1661–1668. 16133966 10.1007/s10620-005-2912-2

[234] Lidar M, Langevitz P, Barzilai O, Ram M, Porat-Katz BS, et al (2009) Infectious serologies and autoantibodies in inflammatory bowel disease: insinuations at a true pathogenic role. Ann N Y Acad Sci 1173: 640–648. 19758210 10.1111/j.1749-6632.2009.04673.x

[235] Rostami Nejad M, Rostami K, Cheraghipour K, Nazemalhosseini Mojarad E, Volta U, et al (2011) Celiac disease increases the risk of *Toxoplasma gondii* infection in a large cohort of pregnant women. Am J Gastroenterol 106: 548–549. 21378773 10.1038/ajg.2010.425

[236] Alvarado-Esquivel C, Estrada-Martinez S (2011) *Toxoplasma gondii* infection and abdominal hernia: evidence of a new association. Parasit Vectors 4: 112. 21682896 10.1186/1756-3305-4-112 PMC3130683

[237] Bars L, Hecht Y, Callard P, Ferrier JP (1978) [Hepatitis due to acquired toxoplasmosis. Case report and review of the literature]. Med Chir Dig 7: 485–486. 732375

[238] Weitberg AB, Alper JC, Diamond I, Fligiel Z (1979) Acute granulomatous hepatitis in the course of acquired toxoplasmosis. N Engl J Med 300: 1093–1096. 431613 10.1056/NEJM197905103001907

[239] Frenkel JK, Remington JS (1980) Hepatitis in toxoplasmosis. N Engl J Med 302: 178–179. 7350455

[240] Roca B, Calabuig C, Arenas M (1992) [Toxoplasmosis and hepatitis]. Med Clin (Barc) 99: 595–596. 1460919

[241] Vethanyagam A, Bryceson AD (1976) Acquired toxoplasmosis presenting as hepatitis. Trans R Soc Trop Med Hyg 70: 524–525. 841657 10.1016/0035-9203(76)90142-5

[242] Lampon N, Hermida-Cadahia EF, Riveiro A, Tutor JC (2012) Association between butyrylcholinesterase activity and low-grade systemic inflammation. Ann Hepatol 11: 356–363. 22481455

[243] Das UN (2007) Acetylcholinesterase and butyrylcholinesterase as possible markers of low-grade systemic inflammation. Med Sci Monit 13: RA214–221. 18049445

[244] Das UN (2012) Acetylcholinesterase and butyrylcholinesterase as markers of low-grade systemic inflammation. Ann Hepatol 11: 409–411. 22481463

[245] Agmon-Levin N, Ram M, Barzilai O, Porat-Katz BS, Parikman R, et al (2009) Prevalence of hepatitis C serum antibody in autoimmune diseases. J Autoimmun 32: 261–266. 19356903 10.1016/j.jaut.2009.02.017

[246] Pavlov VA (2008) Cholinergic modulation of inflammation. Int J Clin Exp Med 1: 203–212. 19079659 PMC2592596

[247] Bertoli F, Espino M, Arosemena JR, Fishback JL, Frenkel JK (1995) A spectrum in the pathology of toxoplasmosis in patients with acquired immunodeficiency syndrome. Arch Pathol Lab Med 119: 214–224. 7887774

[248] Coash M, Forouhar F, Wu CH, Wu GY (2012) Granulomatous liver diseases: a review. J Formos Med Assoc 111: 3–13. 22333006 10.1016/j.jfma.2011.11.023

[249] Ustun S, Aksoy U, Dagci H, Ersoz G (2004) Incidence of toxoplasmosis in patients with cirrhosis. World J Gastroenterol 10: 452–454. 14760779 10.3748/wjg.v10.i3.452 PMC4724918

[250] Bermudez LE, Covaro G, Remington J (1993) Infection of murine macrophages with *Toxoplasma gondii* is associated with release of transforming growth factor beta and downregulation of expression of tumor necrosis factor receptors. Infect Immun 61: 4126–4130. 8406801 10.1128/iai.61.10.4126-4130.1993 PMC281134

[251] Seabra SH, de Souza W, Damatta RA (2004) *Toxoplasma gondii* exposes phosphatidylserine inducing a TGF-beta1 autocrine effect orchestrating macrophage evasion. Biochem Biophys Res Commun 324: 744–752. 15474490 10.1016/j.bbrc.2004.09.114

[252] Borthwick LA, Wynn TA, Fisher AJ (2013) Cytokine mediated tissue fibrosis. Biochim Biophys Acta 1832: 1049–1060. 23046809 10.1016/j.bbadis.2012.09.014 PMC3787896

[253] Da Silva AS, Tonin AA, Thorstenberg ML, Leal DB, Fighera R, et al (2013) Relationship between butyrylcholinesterase activity and liver injury in mice acute infected with *Toxoplasma gondii* . Pathol Res Pract 209: 95–98. 23313104 10.1016/j.prp.2012.10.007

[254] Shapira Y, Agmon-Levin N, Renaudineau Y, Porat-Katz BS, Barzilai O, et al (2012) Serum markers of infections in patients with primary biliary cirrhosis: evidence of infection burden. Exp Mol Pathol 93: 386–390. 23022373 10.1016/j.yexmp.2012.09.012

[255] Haskell L, Fusco MJ, Ares L, Sublay B (1989) Disseminated toxoplasmosis presenting as symptomatic orchitis and nephrotic syndrome. Am J Med Sci 298: 185–190. 2801755 10.1097/00000441-198909000-00008

[256] Fitzgerald JF (1988) Cholestatic disorders of infancy. Pediatr Clin North Am 35: 357–373. 3278286 10.1016/s0031-3955(16)36435-5

[257] Glassman MS, Dellalzedah S, Beneck D, Seashore JH (1991) Coincidence of congenital toxoplasmosis and biliary atresia in an infant. J Pediatr Gastroenterol Nutr 13: 298–300. 1791508 10.1097/00005176-199110000-00011

[258] de Oliveira Fdos S, Kieling CO, dos Santos JL, de Leon Lima PP, Vieira S, et al (2010) Serum and tissue transforming [corrected] growth factor beta1 in children with biliary atresia. J Pediatr Surg 45: 1784–1790. 20850621 10.1016/j.jpedsurg.2010.04.007

[259] Al-Masri AN, Flemming P, Rodeck B, Melter M, Leonhardt J, et al (2006) Expression of the interferon-induced Mx proteins in biliary atresia. J Pediatr Surg 41: 1139–1143. 16769349 10.1016/j.jpedsurg.2006.02.022

[260] Hayashida M, Nishimoto Y, Matsuura T, Takahashi Y, Kohashi K, et al (2007) The evidence of maternal microchimerism in biliary atresia using fluorescent in situ hybridization. J Pediatr Surg 42: 2097–2101. 18082716 10.1016/j.jpedsurg.2007.08.039

[261] MacSween RN, Galbraith I, Thomas MA, Watkinson G, Ludlam GB (1973) Phytohaemagglutinin (PHA) induced lymphocyte transformation and *Toxoplasma gondii* antibody studies in primary biliary cirrhosis. Evidence of impaired cell-mediated immunity. Clin Exp Immunol 15: 35–42. 4765720 PMC1553872

[262] Prandota J (2013) *T. gondii* infection acquired during pregnancy and/or after birth may be responsible for development of both type 1 and 2 diabetes mellitus. J Diabetes Metab 4: 55.

[263] Gokce C, Yazar S, Bayram F, Gundogan K, Yaman O, et al (2008) Anti-*Toxoplasma gondii* antibodies in type 2 diabetes. Natl Med J India 21: 51–51. 18472707

[264] Krause I, Anaya JM, Fraser A, Barzilai O, Ram M, et al (2009) Anti-infectious antibodies and autoimmune-associated autoantibodies in patients with type I diabetes mellitus and their close family members. Ann N Y Acad Sci 1173: 633–639. 19758209 10.1111/j.1749-6632.2009.04619.x

[265] Slosarkova S, Literak I, Skrivanek M, Svobodova V, Suchy P, et al (1999) Toxoplasmosis and iodine deficiency in Angora goats. Vet Parasitol 81: 89–97. 10030751 10.1016/s0304-4017(98)00244-1

[266] Vasquez-Garibay EM, Romero-Velarde E (2009) Iodine deficiency in relation to iron deficiency and parasitosis: Effect of iron status and parasites on iodine deficiency disorders. Comprehensive Handbook of Iodine Nutritional, Biochemical, Pathological and Therapeutic Aspects: 499–511.

[267] Singh S, Singh N, Pandav R, Pandav CS, Karmarkar MG (1994) *Toxoplasma gondii* infection & its association with iodine deficiency in a residential school in a tribal area of Maharashtra. Indian J Med Res 99: 27–31. 8163298

[268] Marani L, Venturi S (1986) [Iodine and delayed immunity]. Minerva Med 77: 805–809. 3714096

[269] Tozzoli R, Barzilai O, Ram M, Villalta D, Bizzaro N, et al (2008) Infections and autoimmune thyroid diseases: parallel detection of antibodies against pathogens with proteomic technology. Autoimmun Rev 8: 112–115. 18700170 10.1016/j.autrev.2008.07.013

[270] Galofre JC (2012) Microchimerism in graves' disease. J Thyroid Res 2012: 724382. 22577597 10.1155/2012/724382 PMC3337626

[271] Renne C, Ramos Lopez E, Steimle-Grauer SA, Ziolkowski P, Pani MA, et al (2004) Thyroid fetal male microchimerisms in mothers with thyroid disorders: presence of Y-chromosomal immunofluorescence in thyroid-infiltrating lymphocytes is more prevalent in Hashimoto's thyroiditis and Graves' disease than in follicular adenomas. J Clin Endocrinol Metab 89: 5810–5814. 15531546 10.1210/jc.2004-1049

[272] Tomairek HA, Saeid MS, Morsy TA, Michael SA (1982) *Toxoplasma gondii* as a cause of rheumatoid arthritis. J Egypt Soc Parasitol 12: 17–23. 7086217

[273] Mousa MA, Soliman HE, el Shafie MS, Abdel-Baky MS, Aly MM (1988) *Toxoplasma* seropositivity in patients with rheumatoid arthritis. J Egypt Soc Parasitol 18: 345–351. 3373059

[274] Torrey EF, Yolken RH (2001) The schizophrenia-rheumatoid arthritis connection: infectious, immune, or both? Brain Behav Immun 15: 401–410. 11782106 10.1006/brbi.2001.0649

[275] Balleari E, Cutolo M, Accardo S (1991) Adult-onset Still's disease associated to *Toxoplasma gondii* infection. Clin Rheumatol 10: 326–327. 1790646 10.1007/BF02208701

[276] Chan WF, Atkins CJ, Naysmith D, van der Westhuizen N, Woo J, et al (2012) Microchimerism in the rheumatoid nodules of patients with rheumatoid arthritis. Arthritis Rheum 64: 380–388. 21953057 10.1002/art.33358 PMC3258459

[277] Shapira Y, Agmon-Levin N, Selmi C, Petrikova J, Barzilai O, et al (2012) Prevalence of anti-*Toxoplasma* antibodies in patients with autoimmune diseases. J Autoimmun 39: 112–116. 22297145 10.1016/j.jaut.2012.01.001

[278] Behan WM, Behan PO, Draper IT, Williams H (1983) Does *Toxoplasma* cause polymyositis? Report of a case of polymyositis associated with toxoplasmosis and a critical review of the literature. Acta Neuropathol (Berl) 61: 246–252. 6650138 10.1007/BF00691993

[279] Hassene A, Vital A, Anghel A, Guez S, Series C (2008) Acute acquired toxoplasmosis presenting as polymyositis and chorioretinitis in immunocompetent patient. Joint Bone Spine 75: 603–605. 18406191 10.1016/j.jbspin.2007.08.009

[280] Gomes AF, Guimaraes EV, Carvalho L, Correa JR, Mendonca-Lima L, et al (2011) *Toxoplasma gondii* down modulates cadherin expression in skeletal muscle cells inhibiting myogenesis. BMC Microbiol 11: 110. 21592384 10.1186/1471-2180-11-110 PMC3116462

[281] Cuturic M, Hayat GR, Vogler CA, Velasques A (1997) Toxoplasmic polymyositis revisited: case report and review of literature. Neuromuscul Disord 7: 390–396. 9327404 10.1016/s0960-8966(97)00098-9

[282] Karasawa T, Takizawa I, Morita K, Ishibashi H, Kanayama S, et al (1981) Polymyositis and toxoplasmosis. Acta Pathol Jpn 31: 675–680. 7282366 10.1111/j.1440-1827.1981.tb02762.x

[283] Cuomo G, D'Abrosca V, Rizzo V, Nardiello S, La Montagna G, et al (2013) Severe polymyositis due to *Toxoplasma gondii* in an adult immunocompetent patient: a case report and review of the literature. Infection 41: 859–862. 23543435 10.1007/s15010-013-0427-x

[284] Arnson Y, Amital H, Guiducci S, Matucci-Cerinic M, Valentini G, et al (2009) The role of infections in the immunopathogensis of systemic sclerosis—evidence from serological studies. Ann N Y Acad Sci 1173: 627–632. 19758208 10.1111/j.1749-6632.2009.04808.x

[285] Arieli G, Arieli S, Feuerman EJ (1979) [*Toxoplasma* infection in scleroderma]. Harefuah 96: 338. 488826

[286] Wilcox MH, Powell RJ, Pugh SF, Balfour AH (1990) Toxoplasmosis and systemic lupus erythematosus. Ann Rheum Dis 49: 254–257. 2339908 10.1136/ard.49.4.254 PMC1004049

[287] Lidar M, Lipschitz N, Langevitz P, Barzilai O, Ram M, et al (2009) Infectious serologies and autoantibodies in Wegener's granulomatosis and other vasculitides: novel associations disclosed using the Rad BioPlex 2200. Ann N Y Acad Sci 1173: 649–657. 19758211 10.1111/j.1749-6632.2009.04641.x

[288] Smith JR, Cunningham ET Jr (2002) Atypical presentations of ocular toxoplasmosis. Curr Opin Ophthalmol 13: 387–392. 12441842 10.1097/00055735-200212000-00008

[289] Suhardjo, Utomo PT, Agni AN (2003) Clinical manifestations of ocular toxoplasmosis in Yogyakarta, Indonesia: a clinical review of 173 cases. Southeast Asian J Trop Med Public Health 34: 291–297. 12971552

[290] Pleyer U, Torun N, Liesenfeld O (2007) [Ocular toxoplasmosis]. Ophthalmologe 104: : 603–615, quiz 616. 10.1007/s00347-007-1535-8 17530262

[291] Soares JA, Carvalho SF, Caldeira AP (2012) Profile of pregnant women and children treated at a reference center for congenital toxoplasmosis in the northern state of Minas Gerais, Brazil. Rev Soc Bras Med Trop 45: 55–59. 22370829 10.1590/s0037-86822012000100011

[292] Sheets CW, Grewal DS, Greenfield DS (2009) Ocular toxoplasmosis presenting with focal retinal nerve fiber atrophy simulating glaucoma. J Glaucoma 18: 129–131. 19225349 10.1097/IJG.0b013e318179f83f PMC2702091

[293] Orefice JL, Costa RA, Orefice F, Campos W, da Costa-Lima D Jr, et al (2007) Vitreoretinal morphology in active ocular toxoplasmosis: a prospective study by optical coherence tomography. Br J Ophthalmol 91: 773–780. 17135336 10.1136/bjo.2006.108068 PMC1955597

[294] Stahl W, Dias JA, Turek G, Kaneda Y (1995) Etiology of ovarian dysfunction in chronic murine toxoplasmosis. Parasitol Res 81: 114–120. 7731917 10.1007/BF00931615

[295] Furtado GC, Cao Y, Joiner KA (1992) Laminin on *Toxoplasma gondii* mediates parasite binding to the beta 1 integrin receptor alpha 6 beta 1 on human foreskin fibroblasts and Chinese hamster ovary cells. Infect Immun 60: 4925–4931. 1399003 10.1128/iai.60.11.4925-4931.1992 PMC258249

[296] Abdoli A, Dalimi A, Movahedin M (2012) Impaired reproductive function of male rats infected with *Toxoplasma gondii* . Andrologia 44: 679–687. 22098674 10.1111/j.1439-0272.2011.01249.x

[297] Stahl W, Kaneda Y, Tanabe M, Kumar SA (1995) Uterine atrophy in chronic murine toxoplasmosis due to ovarian dysfunction. Parasitol Res 81: 109–113. 7731916 10.1007/BF00931614

[298] Terpsidis KI, Papazahariadou MG, Taitzoglou IA, Papaioannou NG, Georgiadis MP, et al (2009) *Toxoplasma gondii*: reproductive parameters in experimentally infected male rats. Exp Parasitol 121: 238–241. 19063884 10.1016/j.exppara.2008.11.006

[299] Lopes WD, da Costa AJ, Santana LF, Dos Santos RS, Rossanese WM, et al. (2009) Aspects of *Toxoplasma* infection on the reproductive system of experimentally infected rams (ovis aries). J Parasitol Res 2009 : Article ID 602803, 6 pages. 10.1155/2009/602803 PMC2915749 20721328

[300] Lopes WDZ, Santos TR, Luvizotto MCR, Sakamoto CAM, Oliveira GP, et al (2011) Histopathology of the reproductive system of male sheep experimentally infected with *Toxoplasma gondii* . Parasitol Res 109: 405–409. 21286752 10.1007/s00436-011-2268-9

[301] Toporovski J, Romano S, Hartmann S, Benini W (2012) Chieffi PP (2012) Nephrotic syndrome associated with toxoplasmosis. Report of seven cases Rev Inst Med Trop Sao Paulo 54: 61–64. 22499417 10.1590/s0036-46652012000200001

[302] Oseroff A (1988) Toxoplasmosis associated with nephrotic syndrome in an adult. South Med J 81: , 95–96. 10.1097/00007611-198801000-00022 3336807

[303] Shahin B, Papadopoulou ZL, Jenis EH (1974) Congenital nephrotic syndrome associated with congenital toxoplasmosis. J Pediatr 85: 366–370. 4610418 10.1016/s0022-3476(74)80117-4

[304] Massiere JP, Delafaye C, Le Guen E, Condat D (1989) [Acquired toxoplasmosis associated with nephrotic syndrome in an adult]. Presse Med 18: 1393. 2529513

[305] Wickbom B, Winberg J (1972) Coincidence of congenital toxoplasmosis and acute nephritis with nephrotic syndrome. Acta Paediatr Scand 61: 470–472. 5041394 10.1111/j.1651-2227.1972.tb15866.x

[306] Beale MG, Strayer DS, Kissane JM, Robson AM (1979) Congenital glomerulosclerosis and nephrotic syndrome in two infants. Speculations and pathogenesis. Am J Dis Child 133: 842–845. 463838 10.1001/archpedi.1979.02130080082017

[307] Pawlowska-Kamieniak A, Mroczkowska-Juchkiewicz A, Papierkowski A (1998) [Henoch-Schoenlein purpura and toxocarosis]. Pol Merkur Lekarski 4: 217–218. 9771003

[308] Hamidou MA, Gueglio B, Cassagneau E, Trewick D, Grolleau JY (1999) Henoch-Schonlein purpura associated with *Toxocara canis* infection. J Rheumatol 26: 443–445. 9972983

[309] Lam C, Imundo L, Hirsch D, Yu Z, D'Agati V (1999) Glomerulonephritis in a neonate with atypical congenital lupus and toxoplasmosis. Pediatr Nephrol 13: 850–853. 10603135 10.1007/s004670050714

[310] Van Velthuysen ML (1996) Glomerulopathy associated with parasitic infections. Parasitol Today 12: 102–107. 15275239 10.1016/0169-4758(96)80669-7

[311] van Velthuysen ML, Florquin S (2000) Glomerulopathy associated with parasitic infections. Clin Microbiol Rev 13: 55–66. 10627491 10.1128/cmr.13.1.55-66.2000 PMC88933

[312] Kapoor S (2012) The close relationship between toxoplasmosis and kidney function Rev Inst Med Trop Sao Paulo. 54: 318–318. 10.1590/s0036-46652012000600011 23152315

[313] Milovanovic I, Vujanic M, Klun I, Bobic B, Nikolic A, et al (2009) *Toxoplasma gondii* infection induces lipid metabolism alterations in the murine host. Mem Inst Oswaldo Cruz 104: 175–178. 19430640 10.1590/s0074-02762009000200008

[314] Coppens I (2006) Contribution of host lipids to *Toxoplasma* pathogenesis. Cell Microbiol 8: 1–9. 16367861 10.1111/j.1462-5822.2005.00647.x

[315] Coppens I, Sinai AP, Joiner KA (2000) *Toxoplasma gondii* exploits host low-density lipoprotein receptor-mediated endocytosis for cholesterol acquisition. J Cell Biol 149: 167–180. 10747095 10.1083/jcb.149.1.167 PMC2175092

[316] Charron AJ, Sibley LD (2002) Host cells: mobilizable lipid resources for the intracellular parasite *Toxoplasma gondii* . J Cell Sci 115: 3049–3059. 12118061 10.1242/jcs.115.15.3049

[317] Sehgal A, Bettiol S, Pypaert M, Wenk MR, Kaasch A, et al (2005) Peculiarities of host cholesterol transport to the unique intracellular vacuole containing Toxoplasma. Traffic 6: 1125–1141. 16262724 10.1111/j.1600-0854.2005.00348.x

[318] Calderon-Margalit R, Adler B, Abramson JH, Gofin J, Kark JD (2006) Butyrylcholinesterase activity, cardiovascular risk factors, and mortality in middle-aged and elderly men and women in Jerusalem. Clin Chem 52: 845–852. 16527886 10.1373/clinchem.2005.059857

[319] Webster JP, Lamberton PHL, Donnelly CA, Torrey EF (2006) Parasites as causative agents of human affective disorders? The impact of anti-psychotic, mood-stabilizer and anti-parasite medication on *Toxoplasma gondii* 's ability to alter host behaviour. Proc R Soc Biol Sci Ser B 273: 1023–1030. 10.1098/rspb.2005.3413 PMC1560245 16627289

[320] Berdoy M, Webster JP, Macdonald DW (2000) Fatal attraction in rats infected with *Toxoplasma gondii* . Proc R Soc Biol Sci Ser B 267: 1591–1594. 10.1098/rspb.2000.1182 PMC1690701 11007336

[321] Flegr J, Kodym P, Tolarová V (2000) Correlation of duration of latent *Toxoplasma gondii* infection with personality changes in women. Biol Psychol 53: 57–68. 10876065 10.1016/s0301-0511(00)00034-x

[322] Dama MS (2012) Parasite stress predicts offspring sex ratio. PLoS ONE 7.. 10.1371/journal.pone.0046169 PMC3458865 23049967

[323] WHO (2008) The Global Burden of Disease: 2004 update. Geneva: World Health Organization.

[324] Lopez AD (2006) Global burden of disease and risk factors. New York, NY, Washington, DC: Oxford University Press, World Bank. 475 p.

[325] Barber N (2004) Sex ratio at birth, polygyny, and fertility: a cross-national study. Soc Biol 51: 71–77. 17019835 10.1080/19485565.2004.9989084

[326] Siegel S, Castellan NJ (1988) Nonparametric statistics for the behavioral sciences. New York: McGraw-Hill. xxiii, 399 p.

[327] Kaňková Š, Kodym P, Flegr J (2011) Direct evidence of *Toxoplasma*-induced changes in serum testosterone in mice. Exp Parasitol 128: 181–183. 21458453 10.1016/j.exppara.2011.03.014

[328] Tabachnick BG, Fidell LS (2007) Using multivariate statistics. Boston: Pearson/Allyn & Bacon. xxviii, 980 p. p.

[329] Thompson B (2006) Foundations of behavioral statistics: an insight-based approach. New York: Guilford Press. xii, 457 p. p.

[330] Fond G, Capdevielle D, Macgregor A, Attal J, Larue A, et al (2013) *Toxoplasma gondii*: a potential role in the genesis of psychiatric disorders. Encephale 39: 38–43. 23095600 10.1016/j.encep.2012.06.014

[331] Flegr J (2013) How and why *Toxoplasma* makes us crazy. Trends Parasitol 29: 156–163. 23433494 10.1016/j.pt.2013.01.007

[332] Vyas A (2013) Parasite-augmented mate choice and reduction in innate fear in rats infected by *Toxoplasma gondii* . J Exp Biol 216: 120–126. 23225874 10.1242/jeb.072983

[333] Hostomská L, Jírovec O, Horáčková M, Hrubcová M (1957) The role of toxoplasmosis in the mother in the development of mongolism in the child (in Czech). Českosl Pediatr 12: 713–723. 13472824

[334] Dimier IH, Bout DT (1998) Interferon-gamma-activated primary enterocytes inhibit *Toxoplasma gondii* replication: a role for intracellular iron. Immunology 94: 488–495. 9767436 10.1046/j.1365-2567.1998.00553.x PMC1364226

[335] Slosarkova S, Literak I, Skrivanek M, Svobodova V, Suchy P, et al (1999) Toxoplasmosis and iodine deficiency in Angora goats. Vet Parasitol 81: 89–97. 10030751 10.1016/s0304-4017(98)00244-1

[336] Kaňková Š, Šulc J, Nouzová K, Fajfrlik K, Frynta D, et al (2007) Women infected with parasite *Toxoplasma* have more sons. Naturwissenschaften 94: 122–127. 17028886 10.1007/s00114-006-0166-2

[337] Kaňkova S, Šulc J, Křivohlavá R, Kuběna A, Flegr J (2012) Slower postnatal motor development in infants of mothers with latent toxoplasmosis during the first 18 months of life. Early Hum Dev 88: 879–884. 22819214 10.1016/j.earlhumdev.2012.07.001

[338] Kaňková Š, Kodym P, Frynta D, Vavřinová R, Kuběna A, et al (2007) Influence of latent toxoplasmosis on the secondary sex ratio in mice. Parasitology 134: 1709–1717. 17651529 10.1017/S0031182007003253

[339] Prandota J (2012) Rhesus-associated glycoprotein (RhAG) phenotype of the red blood cells modulates *T. gondii* infection-associated psychomotor performance reaction times and changes in the human personality profile. Impaired function of the CO_2_, AQP1, and AQP4 gas channels may cause hypoxia and thus enhance neuroinflammation in autistic individuals. In: Gemma C, editor. Neuroinflammation: Pathogenesis, Mechanisms and Management. New York: Nova Publishers. pp. 423–439.

[340] Patja A, Davidkin I, Kurki T, Kallio MJT, Valle M, et al (2000) Serious adverse events after measles-mumps-rubella vaccination during a fourteen-year prospective follow-up. Pediatr Infect Dis J 19: 1127–1134. 11144371 10.1097/00006454-200012000-00002

[341] Packard KA, Khan MM (2003) Effects of histamine on Th1/Th2 cytokine balance. Int Immunopharmacol 3: 909–920. 12810348 10.1016/S1567-5769(02)00235-7

[342] Yazar S, Arman F, Yalcin S, Dimirtas F, Yaman O, et al (2003) Investigation of probable relationship between *Toxoplasma gondii* and cryptogenic epilepsy. Seiz Europ J Epil 12: 107–109. 10.1016/s1059-1311(02)00256-x 12566234

[343] Critchley EM, Vakil SD, Hutchinson DN, Taylor P (1982) *Toxoplasma*, *Toxocara*, and epilepsy. Epilepsia 23: 315–321. 7084141 10.1111/j.1528-1157.1982.tb06197.x

[344] Fuks JM, Arrighi RB, Weidner JM, Kumar Mendu S, Jin Z, et al (2012) GABAergic signaling is linked to a hypermigratory phenotype in dendritic cells infected by *Toxoplasma gondii* . PLoS Pathog 8: e1003051. 23236276 10.1371/journal.ppat.1003051 PMC3516538

[345] Hwang IY, Quan JH, Ahn MH, Ahmed HAH, Cha GH, et al (2010) *Toxoplasma gondii* infection inhibits the mitochondrial apoptosis through induction of Bcl-2 and HSP70. Parasitol Res 107: 1313–1321. 20680337 10.1007/s00436-010-1999-3

[346] Hippe D, Weber A, Zhou LY, Chang DC, Hacker G, et al (2009) *Toxoplasma gondii* infection confers resistance against Bim(S)-induced apoptosis by preventing the activation and mitochondrial targeting of pro-apoptotic Bax. J Cell Sci 122: 3511–3521. 19737817 10.1242/jcs.050963

[347] Hippe D, Lytovchenko O, Schmitz I, Luder CGK (2008) Fas/CD95-mediated apoptosis of type II cells is blocked by *Toxoplasma gondii* primarily via interference with the mitochondrial amplification loop. Infect Immun 76: 2905–2912. 18411295 10.1128/IAI.01546-07 PMC2446730

[348] Kim JY, Ahn MH, Jun HS, Jung JW, Ryu JS, et al (2006) *Toxoplasma gondii* inhibits apoptosis in infected cells by caspase inactivation and NF-kappa B activation. Yonsei Med J 47: 862–869. 17191317 10.3349/ymj.2006.47.6.862 PMC2687828

[349] Hippe D, Vutova P, Hacker G, Gross U, Luder CGK (2006) *Toxoplasma gondii* inhibits host cell apoptosis triggered by both intrinsic and extrinsic proapoptotic signals. Int J Med Microbiol 296: 105–106.

[350] Lim A, Kumar V, Hari Dass SA, Vyas A (2013) *Toxoplasma gondii* infection enhances testicular steroidogenesis in rats. Mol Ecol 22: 102–110. 23190313 10.1111/mec.12042

[351] Flegr J, Lindová J, Pivoňková V, Havlíček J (2008) Brief Communication: latent toxoplasmosis and salivary testosterone concentration-important confounding factors in second to fourth digit ratio studies. Am J Phys Anthropol 137: 479–484. 18615572 10.1002/ajpa.20888

[352] Flegr J, Lindová J, Kodym P (2008) Sex-dependent toxoplasmosis-associated differences in testosterone concentration in humans. Parasitology 135: 427–431. 18205984 10.1017/S0031182007004064

[353] Hollander E, Stein DJ, Kwon JH, Rowland C, Wong CM, et al. (1998) Psychosocial function and economic costs of obsessive–compulsive disorder. CNS Spectrum 3 : 48–50 suppl.

[354] Torres AR, Prince MJ, Bebbington PE, Bhugra D, Brugha TS, et al (2006) Obsessive-compulsive disorder: Prevalence, comorbidity, impact, and help-seeking in the British National Psychiatric Morbidity Survey of 2000. Am J Psychiatry 163: 1978–1985. 17074950 10.1176/ajp.2006.163.11.1978

[355] Kamath P, Reddy YC, Kandavel T (2007) Suicidal behavior in obsessive-compulsive disorder. J Clin Psychiatry 68: 1741–1750. 18052568 10.4088/jcp.v68n1114

[356] Diaconu G, Turecki G (2009) Obsessive-compulsive personality disorder and suicidal behavior: evidence for a positive association in a sample of depressed patients. J Clin Psychiatry 70: 1551–1556. 19607764 10.4088/JCP.08m04636

[357] Wasserman EE, Nelson K, Rose NR, Rhode C, Pillion JP, et al (2009) Infection and thyroid autoimmunity: A seroepidemiologic study of TPOaAb. Autoimmunity 42: 439–446. 19811261 10.1080/08916930902787716

[358] Berlin T, Zandman-Goddard G, Blank M, Matthias T, Pfeiffer S, et al (2007) Autoantibodies in nonautoimmune individuals during infections. Ann N Y Acad Sci 1108: 584–593. 17894023 10.1196/annals.1422.061

[359] Appenzeller S, Shoenfeld Y, de Carvalho JF (2012) Neurologic manifestations of autoimmune diseases. Autoimmune Dis 2012: 683212. 23304458 10.1155/2012/683212 PMC3529895

[360] Fugazzola L, Cirello V, Beck-Peccoz P (2011) Fetal microchimerism as an explanation of disease. Nat Rev Endocrinol 7: 89–97. 21178998 10.1038/nrendo.2010.216

[361] Prandota J (2012) Increased generation of antibodies and autoantibodies directed against brain proteins in patients with autism and their families may be caused by *T. gondii* infection. Maternal and fetal microchimerisms probably play an important role in these processes acting as a “Trojan horse” in dissemination of the parasite. In: Gemma C, editor. Neuroinflammation Pathogenesis, Mechanisms, and Management. New York: Nova Publishers. pp. 447–638.

[362] Wilson GS (1967) Indirect effects: provocation disease. The Hazards of Immunization. London, England: The Athlone Press. pp. 265–280.

[363] Prandota J (2004) Urinary tract diseases revealed after DTP vaccination in infants and young children. Cytokine irregularities and down-regulation of cytochrome P-450 enzymes induced by the vaccine may uncover latent diseases in genetically predisposed subjects. Am J Ther 11: 344–353. 15356430 10.1097/01.mjt.0000117501.97441.c92004

[364] Ansher SS, Thompson W (1994) Modulation of hepatic mRNA levels after administration of lipopolysaccharide and diphtheria and tetanus toxoids and pertussis vaccine adsorbed (DTP vaccine) to mice. Hepatology 20: 984–991. 7523268 10.1002/hep.1840200430

[365] Fantuzzi G, Sironi M, Delgado R, Cantoni L, Rizzardini M, et al (1994) Depression of liver metabolism and induction of cytokine release by diphtheria and tetanus toxoids and pertussis vaccines: role of *Bordetella pertussis* cells in toxicity. Infect Immun 62: 29–32. 8262641 10.1128/iai.62.1.29-32.1994 PMC186063

[366] King MD, Lindsay DS, Holladay S, Ehrich M (2003) Neurotoxicity and immunotoxicity assessment in CBA/J mice with chronic *Toxoplasma gondii* infection and single-dose exposure to methylmercury. Int J Toxicol 22: 53–61. 12573950 10.1080/10915810305075

[367] Pappas G, Roussos N, Falagas ME (2009) Toxoplasmosis snapshots: Global status of *Toxoplasma gondii* seroprevalence and implications for pregnancy and congenital toxoplasmosis. Int J Parasitol 39: 1385–1394. 19433092 10.1016/j.ijpara.2009.04.003

[368] Prandota J (2004) Possible pathomechanisms of sudden infant death syndrome: key role of chronic hypoxia, infection/inflammation states, cytokine irregularities, and metabolic trauma in genetically predisposed infants. Am J Ther 11: 517–546. 15543094 10.1097/01.mjt.0000140648.30948.bd

[369] McMartin KI, Platt MS, Hackman R, Klein J, Smialek JE, et al (2002) Lung tissue concentrations of nicotine in sudden infant death syndrome (SIDS). J Pediatr 140: 205–209. 11865272 10.1067/mpd.2002.121937

[370] Milerad J, Vege A, Opdal SH, Rognum TO (1998) Objective measurements of nicotine exposure in victims of sudden infant death syndrome and in other unexpected child deaths. J Pediatr 133: 232–236. 9709711 10.1016/s0022-3476(98)70225-2

[371] Arling TA, Yolken RH, Lapidus M, Langenberg P, Dickerson FB, et al (2009) *Toxoplasma gondii* antibody titers and history of suicide attempts in patients with recurrent mood disorders. J Nerv Ment Dis 197: 905–908. 20010026 10.1097/NMD.0b013e3181c29a23

[372] Flegr J, Klose J, Novotná M, Berenreitterová M, Havlíček J (2009) Increased incidence of traffic accidents in *Toxoplasma*-infected military drivers and protective effect RhD molecule revealed by a large-scale prospective cohort study. BMC Infect Dis 9: art. 72. 10.1186/1471-2334-9-72 PMC2692860 19470165

[373] Novotná M, Havlíček J, Smith AP, Kolbeková P, Skallová A, et al (2008) *Toxoplasma* and reaction time: Role of toxoplasmosis in the origin, preservation and geographical distribution of Rh blood group polymorphism. Parasitology 135: 1253–1261. 18752708 10.1017/S003118200800485X

[374] Flegr J, Novotná M, Lindová J, Havlíček J (2008) Neurophysiological effect of the Rh factor. Protective role of the RhD molecule against *Toxoplasma*-induced impairment of reaction times in women. Neuroendocrinol Lett 29: 475–481. 18766148

[375] Flegr J, Hampl R, Černochová D, Preiss M, Bičíkova M, et al (2012) The relation of cortisol and sex hormone levels to results of psychological, performance, IQ and memory tests in military men and women. Neuroendocrinol Lett 33: 224–235. 22592206

[376] Overfield J, Hamer D, Dawson M (2007) Introduction to the Rh blood group system. Oxodshire, U.K.: Scion Publishing.

